# Insights Into the Ecological Role of *Pseudomonas* spp. in an Ant-plant Symbiosis

**DOI:** 10.3389/fmicb.2021.621274

**Published:** 2021-02-01

**Authors:** Taise T. H. Fukuda, Camila F. Pereira, Weilan G. P. Melo, Carla Menegatti, Paulo H. M. Andrade, Milton Groppo, Paulo T. Lacava, Cameron R. Currie, Mônica T. Pupo

**Affiliations:** ^1^School of Pharmaceutical Sciences of Ribeirão Preto, University of São Paulo, Ribeirão Preto, Brazil; ^2^Laboratory of Microbiology and Biomolecules, Department of Morphology and Pathology, Center for Biological and Health Sciences, Federal University of São Carlos, São Carlos, Brazil; ^3^Laboratory of Plant Systematics, Department of Biology, Faculty of Philosophy, Sciences and Letters at Ribeirão Preto, University of São Paulo, Ribeirão Preto, Brazil; ^4^Department of Bacteriology, University of Wisconsin-Madison, Madison, WI, United States

**Keywords:** myrmecophytes, bacteria, antimicrobial activities, nitrogen fixation, genome mining, *Pseudomonas*, *Cecropia*, *Azteca* ants

## Abstract

In the myrmecophytic mutualistic relationship between *Aztec*a ants and *Cecropia* plants both species receive protection and exchange nutrients. The presence of microorganisms in this symbiotic system has been reported, and the symbiotic role of some fungi involved in the myrmecophytic interactions has been described. In this work we focus on bacteria within this mutualism, conducting isolations and screening for antimicrobial activities, genome sequencing, and biochemical characterization. We show that *Pantoea*, *Rhizobium*, *Methylobacterium*, *Streptomyces* and *Pseudomonas* are the most common cultivable genera of bacteria. Interestingly, *Pseudomonas* spp. isolates showed potent activity against 83% of the pathogens tested in our antimicrobial activity assays, including a phytopathogenic fungus isolated from *Cecropia* samples. Given the predicted nitrogen limitations associated with the fungal patches within this myrmecophyte, we performed nitrogen fixation analyses on the bacterial isolates within the Proteobacteria and show the potential for nitrogen fixation in *Pseudomonas* strains. The genome of one *Pseudomonas* strain was sequenced and analyzed. The gene cluster involved in the biosynthesis of cyclic lipodepsipeptides (CLPs) was identified, and we found mutations that may be related to the loss of function in the dual epimerization/condensation domains. The compound was isolated, and its structure was determined, corresponding to the antifungal viscosinamide. Our findings of diazotrophy and production of viscosinamide in multiple *Pseudomonas* isolates suggests that this bacterial genus may play an important role in the *Cecropia-Azteca* symbiosis.

## Introduction

Myrmecophytic interactions are mutualistic relationships between ants and plants, in which nutrient exchanges and protection of both species are involved (Fonseca, 1999; Webber et al., 2007; Dejean et al., 2012). Some of these interactions are well studied, such as *Acacia-Pseudomyrmex*, *Tachigali-Pseudomyrmex, Leonardoxa-Petalomyrmex*, *Piper*-*Pheidole*, *Macaranga*-*Crematogaster, Cordia-Allomerus*, and *Cecropia-Azteca* (Heil and McKey, 2003; Voglmayr et al., 2011; González-Teuber et al., 2014; Mayer et al., 2014; Hernández et al., 2017). Myrmecophytes offer shelters (hollow stems that provide nesting space) and food to associated ants through the production of structures such as glycogen-rich Müllerian corpuscles, pearl bodies, and extrafloral nectaries (Davidson and Fisher, 1991; Oliveira et al., 2015). In return, ants assist the host plant in defense against different kinds of enemies as parasitic plants, sap-sucking insects, competitors, pathogens, and a broad range of herbivores (Heil and McKey, 2003; Dejean et al., 2012).

The neotropical trees of the genus *Cecropia* (Cecropiaceae) are abundant and iconic examples of ant-plant mutualism (Heil and McKey, 2003; Oliveira et al., 2015; Treiber et al., 2016). Most myrmecophytic *Cecropia* species are associated with ants from the *Azteca* genus (Formicidae: Dolichoderinae). These ants comprise about 150 different described species found only in tropical regions (Ayala et al., 1996; Berg et al., 2005), and have evolved to be obligately associated with their mutualistic plants (Longino, 1991; Oliveira et al., 2015).

The symbiosis involving *Cecropia* plants and *Azteca* ants encompasses other species. Fungal mycelia are found inside the domatia, in contact with the plant; however, they do not invade the plant tissue. These fungi are classified as black yeasts belonging to the order Chaetothyriales (Mayer and Voglmayr, 2009; Voglmayr et al., 2011; Nepel et al., 2016; Pringle and Moreau, 2017). The larvae of the symbiotic ant ingest the fungus, indicating a nutritive function (Blatrix et al., 2012).

Proteobacteria and Actinobacteria associated with ant-plant systems have been reported and may be involved in different myrmecophyte interactions (Hanshew et al., 2015; Lucas et al., 2019; Ruiz-González et al., 2019). The microbial communities present inside the domatia of ant-plant symbioses appear to be influenced by ant species and, to a lower extent, by the local environment (Ruiz-González et al., 2019). The presence of these microbes and the potential that ants actively select for at least part of the associated microorganisms suggests that bacteria could play beneficial roles in the survival of the colonies and their host plants.

The bacterium *Pseudomonas citronellolis* (Gamma proteobacteria: Pseudomonadales) was recently proposed to be specifically associated with *Azteca* ants that engage in myrmecophyte interaction (Ruiz-González et al., 2019). Other bacteria including *Burkholderia*, *Enterobacter*, *Pantoea*, and *Rhizobium* were also found in *Cecropia-Azteca*, and it is believed that they may perform different roles, such as defense against parasites, plant substrate degradation, and nitrogen-fixing as described for fungus-growing ants (Pinto-Tomás et al., 2009; Zucchi et al., 2011; Lucas et al., 2019; Ruiz-González et al., 2019).

Although the *Cecropia-Azteca* mutualism has been described for more than a hundred years, there are few studies on the microbiome of this environment and the chemical compounds that mediate the interaction of present organisms. Here, we investigated the presence of bacteria involved in the *Cecropia-Azteca* interaction, seeking to understand the ecological role of these microorganisms within this symbiosis as well as exploring such strains as promising sources of antimicrobial compounds.

## Materials and Methods

### Strains Isolation and Identification

The research and collection of biological samples was authorized by the Brazilian government (SISBIO authorization 46555-6, CNPq process 010936/2014-9). Collections were performed in October 2015 and December 2018 in the Atlantic Forest at Itatiaia National Park, RJ, Brazil. GPS coordinates, information of isolated bacteria and *Cecropia* trees are in Supplementary Table 1. *Cecropia* species were identified using Gaglioti and Romaniuc-Neto (2012), and Brazil Flora Group (BFG) (2015). Vouchers were deposited at the Herbarium of the Department of Biology, FFCLRP-USP (Herbarium SPFR).

Samples of *Cecropia* were collected from the internodal space that was associated with *Azteca* ants. Fungal patches samples, worker ants, pupae, and larvae were also collected. For the isolation of bacteria, parts of the collected samples were placed in Petri dishes containing ISP-2 medium (4 g of yeast extract, 4 g of dextrose, 10 g of malt extract and 20 g of agar, and 1 L of distilled water), supplemented with the antifungals nystatin (40 mg/L) and cycloheximide (50 mg/L). Plates were incubated at 30°C and the appearance of colonies was monitored for four weeks. For the isolation of actinobacteria, fragments of the collected samples were placed on chitin plates (in one liter: 5.33 g of chitin, 0.767 g K_2_HPO_4_, 0.367 g KH_2_PO_4_, 0.244 g MgSO_4_, 0.01 g FeSO_4_⋅7H_2_O, 0.001 g ZnSO_4_⋅ 7H_2_O, 0.001 g MnCl_2_ ⋅ 4H_2_O, 20 g of agar) supplemented with the antifungals nystatin (40 mg/L) and cycloheximide (50 mg/L) (Hsu and Lockwood, 1975). After four weeks of growth at 30°C, bacterial colonies were subcultured onto ISP-2-agar plates, and serial culturing was done until the obtention of pure cultures.

### DNA Extraction From Microorganisms

The bacterial strains were grown in ISP-2 medium and incubated at a controlled temperature of 30°C for 2–3 days. After this the DNA extraction of the bacteria was carried out using the microLYSIS^®^-Plus solution according to the manufacturer’s instructions.

Genomic DNA from actinobacteria was extracted using an adapted protocol (Kumar et al., 2010). Strains were grown in Tryptone Soy Broth (BD, United States) at 30°C and 300 rpm for 3–5 days. After this period, the culture was transferred to Eppendorf microtubes and centrifuged at 10,000 × *g* for 10 min. The supernatant was removed, and the pellet frozen at -20°C. The pellet was washed in 500 μL of sucrose 10.3%, centrifuged for 1 min at 10,000 × g, and the supernatant was discarded. Then 450 μL of TSE (50% Sucrose; 0.5 M EDTA pH 8 and 1 M Tris Buffer pH 8) were added with lysozyme and incubated for 20 min at 37°C. After that period, 13 μL of proteinase K were added and incubated for another 15 min at 55°C. At the end of the incubation time, 250 μL of 2% SDS was added, and then gently stirred until the formation of a clear solution was observed. Then, 300 μL of phenol: chloroform pH 8.0 were added, mixed, and then centrifuged for 10 min at 4°C. The supernatant was transferred to another tube, and 60 μL of 3M NaOAc, pH 6.0, and 700 μL of isopropanol were added. The contents were mixed until the appearance of “white strings”. The tube was centrifuged down briefly and poured off the supernatant. The DNA was resuspended in 100–200 μL of autoclaved deionized H_2_O and quantified using NanoDrop^®^.

### 16S rRNA Sequencing

PCR amplification of the 16S rRNA gene of bacteria were performed using two primers: 27F (5′-AGAGTTTGATCMT GGCT-3′) and 1492R (5′-TACGGYTACCTTGTTACGACTT-3′). The final reaction volume of 15 μL contained: 8 μL EconoTac^®^ DNA Polymerase (Lucigen, United States), 0.5 μL of each primer 27F and 1492R, 0.5 μL DMSO, 4.5 μL deionized H_2_O, and 1 μL DNA (10 ng/μL). An initial denaturation step at 94°C for 3 min was followed by 32 cycles of amplification of 94°C for 30 s, 60°C for 30 s, and 72°C for 2 min, and a final extension step of 72°C for 5 min. Amplicons were detected by agarose gel electrophoresis and visualized by ultraviolet (UV) fluorescence after staining with ethidium bromide. The Big Dye sequencing was done using 1.5 μL 5X buffer, 1 μL primer (10 μM), 1 μL BigDye 3.1 (Applied Biosystems), 0.5 μL DMSO, 1 μL PCR product DNA, and deionized water to make up the total volume of 10 μL. The reaction started with 95°C for 3 min, followed by 35 cycles of 96°C for 10 s, 58°C for 3 min and a final extent of 72°C for 7 min. The sequencing reaction was purified with the Axyprep Mag Dyeclean purification kit (Axygen). The DNA was resuspended in 25 μL of deionized H_2_O before submission to the Center for Genetics and Biotechnology at the University of Wisconsin (Biotech Center). The 16S rRNA sequences were searched for homologous sequences with BLASTn in the NCBI database. The sequences are deposited at NCBI GenBank under Accession numbers: MW139977 - MW140014.

### ITS Sequencing

For a strain of the fungus *Pestalotiopsis clavispora* FB1 isolated from *Cecropia* leaves (Supplementary Figure 1) the ITS region (internal transcribed spacer) was amplified using the primers TS1 (5′-TCCGTAGGTGAACCTGCGG-3′) and ITS4 (5′-TCCTCCGCTTATTGATATGC-3′) (White et al., 1990). The PCR consisted of 4.0 μL of dNTPs (1.25 mM each), 2.5 μL of buffer 10X, 1.0 μL of MgCl_2_ (50 mM), 0.2 μL de Taq-polymerase (5.0 U/μL), 2,0 μL of each primer (10 μM), 10.3 μL of ultrapure water and 2.0 μL DNA (10 ng/μL). An initial denaturation step at 94°C for 3 min was followed by 35 cycles of amplification of 94°C for 1 min, 55°C for 1 min and 72°C for 2 min, and a final extension step of 72°C for 5 min. Sequencing was performed as described above for bacteria.

### Genome Sequencing

Genomic DNA from strain *Pseudomonas* sp. ICBG1301 was obtained as described previously in the 16S rRNA extraction from actinobacteria with additional steps. After the appearance of “white strings” it was centrifuged down briefly, poured off the supernatant, and redissolved in 500 μL TE. 10 μL of RNase A (20 mg/mL) were added and rocked at room temperature until dissolved. 300 μL phenol were added and mixed. 150 μL of chloroform were also added and mixed. After that, the sample was spun at 4000 x g for 10 min and the top layer was transferred to a new tube. 300 μL chloroform were added and the solution was spun for 3 min at 4000 × *g*. The top layer was transferred to a tube holding 50 μL 3M NaOAc pH 6 and 350 μL isopropanol, and the tube was inverted until the DNA appeared as a stringy clump. The supernatant was poured off and the DNA was dissolved in deionized H_2_O. The genomic DNA was sequenced by Illumina MiSeq^TM^ System 2 × 300 bp at the Center for Genetics and Biotechnology at the University of Wisconsin (Biotech Center). The assembly method used was SPAdes V3.11.0, the genome coverage was 284.5 X and 29 contigs were obtained. The genome was deposited into Genbank, and its accession number is JAEKGB000000000.

### Phylogenetic Analyses

We used the 16S rRNA gene of the four *Pseudomonas* strains isolated and others present in the NCBI database, that are known to be viscosin-producers, to construct a phylogenetic tree. The sequences were aligned using the MUSCLE alignment algorithm, trimmed manually and the alignments were refined using Geneious Prime 2020.0.4 (https://www.geneious.com). The construction of Maximum-likelihood phylogenetic trees was performed in the IQ-tree 1.6 software using ModelFinder to figure out the best nucleotide replacement model. The TPM3 + F + R2 model was used and branch support was assessed by ultrafast bootstrap UFBoot (1000 bootstrap replicates) (Minh et al., 2020). Phylogenetic tree was visualized with iTol v5 (Letunic and Bork, 2019). The analysis involved 33 nucleotide sequences and the phylogenetic tree was rooted by *Pseudomonas aeruginosa* PAO1.

Four conserved “housekeeping” genes were used for the multilocus sequence analysis (MLSA): 16S rRNA, *gyrB*, *rpoB*, and *rpoD*. All these genes were extracted from the genome of ICBG1301 and 30 other *Pseudomonas* spp. deposited in the NCBI database and the phylogenetic tree was rooted by *Pseudomonas aeruginosa* PAO1.

The sequences belonging to each gene were aligned using MUSCLE, trimmed manually and the alignments were refined using the same algorithm on Geneious Prime 2020.0.4 (https://www.geneious.com). Then the sequences were concatenated. The construction of Maximum-likelihood phylogenetic trees were performed in the IQ-tree 1.6 software using ModelFinder to figure out the best nucleotide replacement model. The TIM + F + I + G4 model was used and branch support was assessed by ultrafast bootstrap UFBoot (1000 bootstrap replicates) (Minh et al., 2020). Phylogenetic tree was visualized with iTol v5 (Letunic and Bork, 2019).

The same method was used for the construction of phylogenetic trees of adenylation and condensation domains. Amino acid sequences of these domains were obtained from the antiSMASH database (Blin et al., 2020). ModelFinder was used in this case to choose the best model for protein evolution.

### Growth of Microorganisms in Nitrogen-free Culture Medium (NFb)

The ability to fix atmospheric nitrogen by the bacteria isolated from *Cecropia-Azteca* symbiosis was evaluated by growing the strains on the semisolid nitrogen-free medium (NFb), composed of malic acid 5 g/L, K_2_HPO_4_ 0.5 g/L, MgSO_4_.7H_2_O 0.2 g/L, NaCl 0.1 g/L, CaCl_2_⋅2H_2_O 0.01 g/L, 2 mL micronutrient solution (CuSO_4_.5H_2_O 0.04 g/L, ZnSO_4_.7H_2_O 1.2 g/L, H_3_BO_3_ 1.4 g/L, Na_2_MoO_4_.2H_2_O 1 g/L and MnSO_4_.H_2_O 1.175 g/L completing the volume to 1 L with distilled H_2_O); 2 mL bromothymol blue (solution 0.5% in 0.2N KOH); 4 mL Fe-EDTA (solution 1.64%); 2 mL vitamin solution (0.1 g/L biotin and 0.02 g/L pyridoxine completing the volume to 1 L with distilled H_2_O); 4.5 g/L KOH; 2.3 g/L agar; make up to 1 L with distilled H_2_O and adjust the pH to 6.8. Glass tubes were filled with 3 mL of the NFb medium, and after solidification, the microorganisms were inoculated in the tube and incubated at 28°C in the dark for 96 hours. Reading was carried out by observing the formation of a growth film close to the surface (Döbereiner et al., 1995; Cattelan et al., 1999; Batista et al., 2018). The strains that showed positive results in this stage were re-inoculated in triplicate to confirm the result.

### Bioactivity Screening

Pairwise interactions against human pathogens (*Staphylococcus aureus* and *Candida albicans*), a phytopathogen (*Rhizopus oryzae*), an entomopathogen (*Metarhizium anisopliae*), and *Bacillus subtilis* were carried out using previously described methods (Getha and Vikineswary, 2002; Visser et al., 2012). A phytopathogen fungus (Lazarotto et al., 2012; Borrero et al., 2018) identified as *Pestalotiopsis clavispora* FB1, isolated from *Cecropia* sp. leaves causing damage to the plant, was also tested. After strains inoculation, plates were maintained at 30°C and monitored for 14 days. Then, we recorded the qualitative result, in which we classified it as “inhibition” or “no inhibition” according to the presence or absence of an inhibition zone.

### Isolation and Structure Determination of Viscosinamide

HPLC purifications were carried out using an HPLC system (Shimadzu, Kyoto, Japan), comprising an LC-6AD solvent pump, a CBM-20A system controller, a CTO-20A column oven, a SIL-20AF injector, a SPD-M20A diode array detector (DAD), a FCR-10A collector, and LabSolutions software for data acquisition. Purification of viscosinamide (**1**) was performed at 3 mL/min with a reversed phase C18 semi-preparative column (Phenomenex Gemini, 10 mm, 250 mm, 5 μm) using a gradient solvent system with aqueous acetonitrile (50% to 100% acetonitrile) for 12 min. The absolute configuration of the amino acids present in the structure was determined using advanced Marfey’s method (Harada et al., 1996). Viscosinamide (**1**) (1 mg) was hydrolyzed at 110°C in 500 μL of 6 N HCl for 24 h. HCl was removed under reduced pressure and the dry material was resuspended in 500 μL of H_2_O and dried three times to remove residual acid. The hydrolyzate was dissolved in 100 μL of 1 N NaHCO_3_ and incubated with 50 μL of 1 mg/mL L-FDLA and L,D-FDLA (FDLA - 2,4-dinitro-5-fluoro-phenyl leucineamide), in acetone, at 80°C for 3 min. The reaction was quenched by adding 50 μL of 2 N HCl. Aqueous acetonitrile (1:1) was added to dissolve the mixture. A 10 μL aliquot of the hydrolyzate derivative was analyzed by HPLC-HR-ESI-MS using an analytical C18 column (Phenomenex Gemini, 4.6 mm, 250 mm, 5 μm), with a gradient solvent system (5% to 100% ACN with 0.1% formic acid over 30 min) and flow rate of 0.7 mL/min. The oven temperature was 35°C. The ionization source parameters were: End Plate Offset voltage, 500V; capillary voltage, 3500 V; nebulizer at 5.5 bar; drying gas flow rate of 10.0 L/min; drying gas temperature, 220°C; for positive mode ionization. The spectra rate of 2.0 Hz and mass range of 300 to 1000 m/z were maintained throughout the analysis. The Data Analysis^®^ Software of Bruker Daltonics instrument was used to analyze the metabolic profile and UV spectrum data.

NMR spectra were recorded using a BRUKER^®^ - DRX500 - Ultra Shield^®^ (^1^H: 500.13 MHz, ^13^C: 125.77 MHz). Spectra were processed using MestReNova 6.0.2. High-resolution MS spectra were obtained using a micrOTOF II-ESI-TOF (Bruker Daltonics^®^), positive ion mode using capillary voltage 3900 V, dry gas flow 4 L h^–1^, and nitrogen as nebulizer gas. Na-TFA 10 mg/mL was used for internal calibration. Samples were prepared at 20 μg/mL, in EtOAc:MeOH:H_2_O (1:2:2). The LC-MS was carried out on a Bruker Daltonics High Pressure Liquid Chromatographer coupled to a diode array UV detector, and a Mass Spectrometer with electrospray ionization source (ESI) and micrOTOF (Time of Flight) detector. MS/MS analysis was performed using the MALDI-TOF/TOF (Bruker Daltonics, UltrafleXtreme, Bremen, Germany). The matrix CHCA (α-cyano-4-hydroxycinnamic acid) was prepared using a saturated solution in aqueous acetonitrile 3:7 (0.1% TFA). The settings of the instrument were: positive ion reflector mode (RV1 e RV2 of 26.60 kV e 13.35 kV, respectively); 500 laser shots per spectrum; PIE (Pulsed ion extraction) of 110 ns, laser frequency of 1000 Hz; IS1 (ion source 1) and IS2 voltages were 25.00 kV e 23.00 kV, respectively; external calibration was carried out using a peptide mixture provided by Bruker. Samples were diluted in methanol and 1 μL of the sample was added to 1 μL of matrix and 1 μL of this mixture was applied to the MALDI plate.

## Results

### Strain Isolation and Identification

**FIGURE 1:**
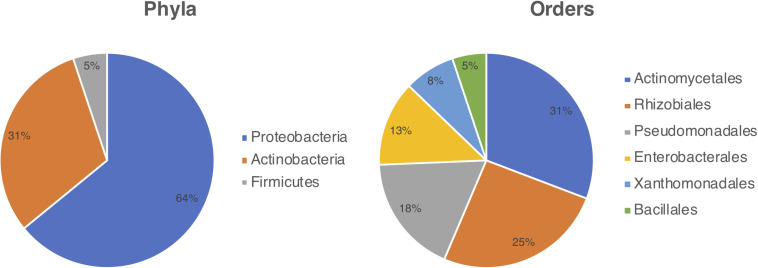
Diversity of bacteria cultured from the *Cecropia-Azteca* symbiosis by bacterial phylum (left) and order (right).

Bacterial strains were isolated from *Cecropia* samples inhabited by *Azteca* ants collected at Itatiaia National Park (RJ, Brazil) in 2015 and 2018. Microbial isolations were performed from various parts of the samples gathered, ranging from ants, eggs, plant parenchyma, and fungal patches found in the hollows of *Cecropia*’s stalks. This process resulted in the isolation of 39 bacterial strains which had their 16S rRNA gene sequenced, allowing their identification at the genus level (Supplementary Table S1). Among the isolates, 25 (64.1%) strains belong to the phylum Proteobacteria, 12 (30.8%) to Actinobacteria, and two (5.1%) to Firmicutes. Six bacterial orders were represented including: Actinomycetales (12), Rhizobiales (10), Pseudomonadales (7), Enterobacterales (5), Xanthomonadales (3), and Bacillales (2) (Figure 1). The isolates belonged to ten different genera namely *Acinetobacter, Actinomadura, Bacillus, Methylobacterium, Pantoea, Pseudomonas, Rhizobium, Raoultella, Stenotrophomonas*, and *Streptomyces*.

Isolates were obtained mainly from *Azteca* ants and fungal patches. *Pseudomonas* spp. and *Rhizobium* spp. were found in three of the four sampled sites (*Cecropia* parenchyma, *Azteca* sp. ant and fungal patches); *Methylobacterium* spp. also in three (*Azteca* sp. ant, *Azteca* sp. egg and *Cecropia* parenchyma); *Pantoea* spp. was found in two (*Azteca* sp. ant and *Azteca* sp. egg); *Bacillus* spp. in two (*Cecropia* parenchyma, fungal patches); *Stenotrophomonas* spp., *Streptomyces* spp. and *Actinomadura* spp. were sampled also in two (*Azteca* sp. ant and fungal patches); *Acinetobacter* spp. and *Raoultella* sp. in just one site (*Azteca* sp. ant and fungal patches, respectively).

### Isolates Growth in Nitrogen-free Medium

Proteobacteria genera *Pantoea*, *Rhizobium*, *Methylobacterium*, and *Pseudomonas* have known diazotrophic potential (Borm et al., 2008; Pinto-Tomás et al., 2009; Ruiz-González et al., 2019; Bar-Shmuel et al., 2020). Due to the significant number of strains isolated from *Cecropia-Azteca* that could potentially fix nitrogen (22; 56.4%), the nitrogenase activity of these strains was tested. All bacteria grew in the nitrogen-free semi-solid NFb media under the tested culture conditions. However, only those belonging to the genus *Pseudomonas* (strains ICBG1870, ICBG1871, ICBG1301, and ICBG1881) showed potential for nitrogen fixation as they formed a white disk near the surface after two re-inoculation rounds. Although some other strains did exhibit growth on nitrogen-free medium they did not show potential to perform biological nitrogen fixation (BNF) as indicated by the re-inoculation step, which is important for confirmation of the result (Cattelan et al., 1999), indicating that their growth may have occurred at the expense of energetic reserves of bacterial cells.

### Biological Activity

Bioassays of the 39 isolated strains were performed against human pathogens, phytopathogens and entomopathogen (Supplementary Figure 2). Sixteen isolates (41%) inhibited at least one of the microorganisms tested, and eight strains (20.5%) inhibited at least two. *Bacillus* spp. strains inhibited the growth of at least three pathogens, including the human pathogen *C. albicans.* The isolates with biological activity against phytopathogens and the entomopathogen were of particular interest since this inhibition could denote ecological relevance. Five out of the 16 strains referred above were active against *P. clavispora*, ten against *R. oryzae*, and seven against *M. anisopliae*. Interestingly, four strains identified as *Pseudomonas* spp. inhibited the growth of almost all microorganisms tested, including the phytopathogen *P. clavispora* FB1 (Supplementary Figure 3).

### *Pseudomonas* sp. ICBG1301 Genome Sequencing and Analysis

According to the biological screening, *Pseudomonas* spp. were the most bioactive bacterial isolates. We selected one strain (ICBG1301) to sequence its genome and analyze the respective biosynthetic gene clusters. Genome analysis of *Pseudomonas* sp. ICBG1301 with antiSMASH 5.0 (Blin et al., 2019) revealed 14 biosynthetic gene clusters (BGCs), encoding the production of siderophore, non-ribosomal peptides, N-acetylglutaminylglutamine amide, beta-lactone, thiopeptide, arylpolyene, and bacteriocins. We therefore decided to investigate BGCs encoding for the production of antifungal compounds. Cluster 9.1 showed 43% similarity to the antifungal non-ribosomal peptide (NRP) viscosin according to the antiSMASH output. NRPs are biosynthesized by specialized enzymes called Non-Ribosomal Peptide Synthetases (NRPS). These NRPS are megaenzymes, which have several catalytic domains, each of which is responsible for a stage of the biosynthesis of the final product. Essential domains are involved in the formation of the mature peptide, called PCP (peptide carrier protein), A (adenylation), C (condensation) and TE (thioesterase termination, usually found only once in a gene cluster) (Finking and Marahiel, 2004). Bioinformatic analyses on these domains can give us insights into the specificities of the monomers recruited and their stereochemistry. In order to evaluate whether the BGC could be responsible for the production of viscosin or not, analyses were performed with its condensation and adenylation domains.

**FIGURE 2:**
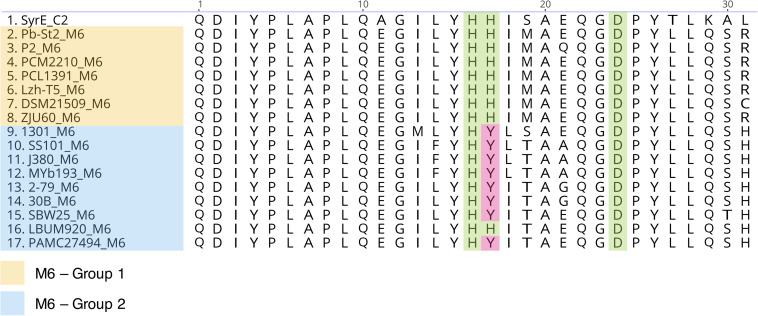
Alignment of the condensation domains (C_dual_) of the sixth module involved in the biosynthesis of viscosin CLPs. The first 30 amino acids of the N-terminal portion are shown. Highlighted amino acids are those described as belonging to the catalytic triad (Balibar et al., 2005).

Phylogenetic analyses of the adenylation domains of cluster 9.1 and other 20 viscosin-producing strains were done and the final prediction of the peptide was obtained based on the substrate specificity of nine modules, being: Leu1-Gln2/Glu2-Thr3-Val4-Leu5-Ser6-Leu-7-Ser8-Ile9. These analyses showed that the adenylation domains responsible for recruiting the same monomer, in the case of modules 6/8 (Ser) and 1/7 (Leu), are subdivided according to the groups observed in Figure 2 (Supplementary Figure 8). The compound encoded by cluster 9.1 belongs to a group – the viscosin group – of cyclic lipodepsipeptides. Members of this group comprise viscosin, viscosinamide, WLIP (White Line-Inducing Principle), pseudodesmin and massetolide (Supplementary Figure 16). These compounds differ from each other in the absolute configuration of one amino acid (Leu5) or in the amino acid composition of the third and/or fourth positions. Adenylation domains were grouped according to the amino acid incorporated, and each one formed two clades. The domains of *Pseudomonas* strains that produce pseudodesmin or WLIP were grouped together, and the domains involved in the biosynthesis of viscosin and massetolide formed another. A specific analysis of *Pseudomonas* sp. ICBG1301 showed that the A domains are grouped with those of *P. fluorescens* SBW25, *P. antarctica* PAMC 27494 and *Pseudomonas* sp. LBUM990. *P. fluorescens* SBW25 produces viscosin, as confirmed by NMR, raising the hypothesis that the ICBG1301 strain also produces viscosin. It could be possible to bioinformatically differentiate viscosin, viscosinamide, WLIP, pseudodesmin and massetolide producers by analyzing the specificity of the adenylation domain of the second module. Strains that show such specificity for glutamic acid could be classified as producers of viscosin, WLIP and massetolide, whereas those that show specificity for glutamine could be producers of viscosinamide and pseudodesmin. However, the structural difference between these two amino acids is not so significant and the adenylation domains are grouped together.

Furthermore, phylogenetic analysis of the condensation domains revealed that, except for modules 1 and 8 (C_*starter*_ and ^*L*^C_*L*_, respectively), all modules contain a C_dual_ domain (Supplementary Figures 5–7). C_dual_ domains are responsible for catalyzing the condensation reaction and epimerization of the upstream amino acid. Intriguingly, domains belonging to the sixth module were split into two different groups (Supplementary Figure 7). Except for one strain (*Pseudomonas* sp. LBUM920), strains that belong to group 2 have a Histidine → Tyrosine mutation in the N-terminal catalytic triad (Figure 2). According to the literature, all viscosin group members show an L-amino acid in the first position. Thus, during the evolution of this pathway, a mutation in the C-domain of the second module has possibly occurred, and this domain has lost its function. Similarly, another mutation might have occurred in the region coding for the sixth module C-domain. This mutation could be responsible for the loss of function of their respective C_dual_ domains. Consequently, it is possible to infer that group 1 strains have the functional domain; thus, they may produce pseudodesmin, showing a D-Leu5, while group 2 strains produce viscosin, showing a L-Leu5. In general, the gene clusters related to viscosin, viscosinamide and massetolide biosynthesis are split into two loci, while the pseudodesmin and WLIP clusters are in contiguous clusters. This observation is consistent with our analyses, since group 1 strains show a contiguous gene cluster and group 2 strains show clusters split into two loci. BGC 9.1 of *Pseudomonas* sp. ICBG1301 is split into two loci, therefore, potentially related to the production of viscosin or viscosinamide.

### *Pseudomonas* spp. Phylogenetic Analysis

**FIGURE 3:**
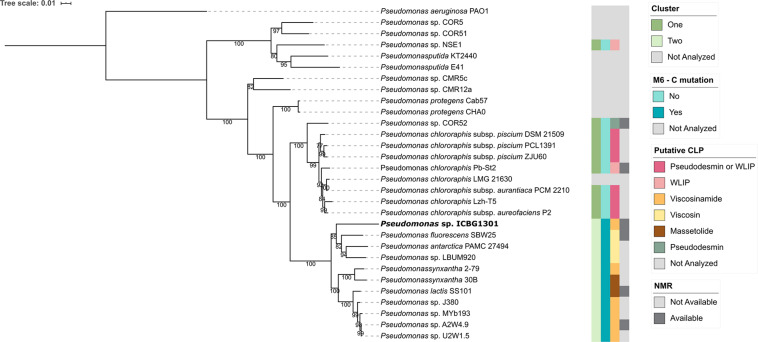
Phylogenetic representation of *Pseudomonas* strains. The phylogeny is based on the concatenated sequence of 16S rRNA, *gyrB, rpoB, and rpoD* genes. The bootstrap support values are based on 1000 bootstrap replicates. The phylogeny was reconstructed using IQ-TREE, using the TIM + F + I + G4 model of nucleotide substitution and including *P. aeruginosa* PAO1 as outgroup. Strains whose BGCs were not analyzed were either due to the absence of a publicly available sequence or they produce CLPs that do not belong to the viscosin class.

After analyzing the gene clusters involved with viscosin biosynthesis, we decided to investigate the evolutionary relationship between *Pseudomonas* strains. We built a Maximum likelihood tree using the 16S rRNA (Supplementary Figure 4) and a multilocus phylogeny based on the 16S rRNA, *gyrB*, *rpoB*, and *rpoD* gene sequences (Figure 3).

It is noteworthy that the strains that showed the mutation in the C6-domain also have the cluster split into two loci and share a common ancestor, forming a monophyletic group. Viscosinamide (*P. synxantha* 2-79, *Pseudomonas* sp. J380, *Pseudomonas* sp. MYb193, *Pseudomonas* sp. A2W4.9 and *Pseudomonas* sp. U2W1.5) and massetolide (*P. synxantha* 30B and *P. lactis* SS101) producers formed a group, apart from the viscosin clade. Viscosinamide production appears either as an ancestor in the group or appearing three times in the evolution of the group. Massetolide appears twice in the evolution of the group and viscosin only once. The most reasonable hypothesis in this clade is the ancestral production of viscosinamide, with later change to massetolide (two evolutionary events) and viscosin (one evolutionary event). Strain ICBG1301 is related to viscosin-producing strains (*P. fluorescens* SBW25, *P. antarctica* PAMC 27494 and *Pseudomonas* sp. LBUM990). This raises the hypothesis that ICBG1301 could either produce viscosin or viscosinamide.

### Viscosinamide

Cultures of *Pseudomonas* spp. ICBG1870 and ICBG1881, isolated from different ant colonies, were scaled up and extracted with ethyl acetate. Rounds of bioguided fractionation led to the isolation of the active compound, with an *m/z* 1147.6976 [M + Na]^+^ (error 2.4 ppm) (Supplementary Figure 9). Both samples showed identical ^1^H NMR profiles, corresponding to the same compound. NMR and MALDI-MS/MS data confirmed the structure of the CLP (Supplementary Table S2, Supplementary Figures 10–14). Signals in the region of δ_H_ 3.44–4.57 were observed in the ^1^H NMR spectrum, which correspond to the α-protons of the amino acids. These α-protons are bonded to carbons at δ_C_ 53.4–65.8, as deduced from *g*HSQC spectrum. The *g*COSY spectrum showed α-protons are coupled with the side chain hydrogens (δ_H_ 1.87, 2.02, 5.36, 2.19, 1.77, 3.79, 1.60, 3.65, and 1.98). The α-protons also correlate with the carbonyl carbons of the adjacent amino acid (δ_C_ 170.1–176.6), as seen in the *g*HMBC spectrum. Hydrogens at δ_H_ 4.04 and 5.11 have no correlations with carbons in *g*HSQC and are directly linked to oxygens in the side chains of Ser6 and Ser8. Other hydrogen signals that do not show correlations in the *g*HSQC spectrum are those linked to the nitrogens of the peptide bonds (δ_H_ 9.23, 8.74, 8.47, 8.25, 8.02, 7.95, 7.47, 7.08, and 6.61). Resonances of CH_2_ hydrogens are observed in the most shielded region of the spectrum signals (δ_H_ 0.7 - 2.6) and correspond to the alkyl chain of the fatty acid.

**FIGURE 4:**
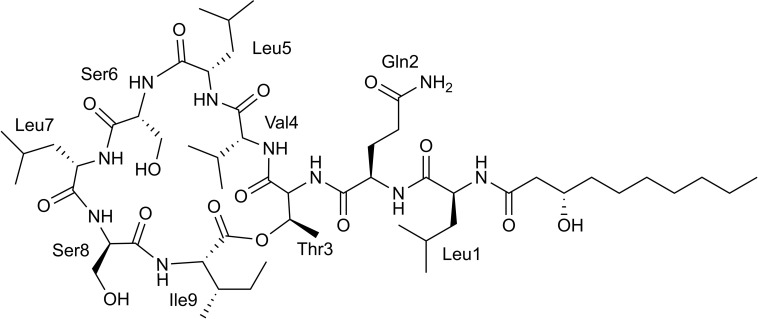
Chemical structure of viscosinamide.

Hydrolysis and advanced Marfey’s derivatization, followed by LC-MS, confirmed the bioinformatic predictions of the stereocenters. The chromatogram shows the presence of D-Glu instead of D-Gln, causing a divergence of one mass unit with the HRESI-MS analysis, due to the conversion of Gln to Glu during the hydrolysis process (Shih, 1985; Li et al., 2010). In addition, the chromatogram showed the presence of only L-Leu in the structure (Supplementary Figure 15), corresponding to the antifungal viscosinamide (**1**) (Figure 4; Nielsen et al., 1999). This compound was also detected by LC-MS in the extract of *Pseudomonas* sp. ICBG1301. This result agrees with the antifungal activity of the producer strains against *M. anisopliae*, *P. clavispora*, and *R. oryzae*.

## Discussion

The occurrence of bacterial strains in the myrmecophytic interactions has been reported (Heil et al., 2010; Lucas et al., 2019; Ruiz-González et al., 2019). Here, we observed that the ant-plant system *Cecropia-Azteca* harbors a rich bacterial community belonging to three phyla: Proteobacteria, Actinobacteria, and Firmicutes. Proteobacteria and Actinobacteria strains correspond to 94.9% of the isolated bacteria. Our results agree with previous studies that analyzed the abundance of the microbial community in the interaction between *Cecropia peltata* and *Azteca alfari.* Although Lucas et al. (2019) identified microorganisms belonging to a higher number of phyla (n = 22), they also found the largest proportion (90%) corresponding to Proteobacteria and Actinobacteria. This difference in the number of phyla isolated in our work could be explained by two factors: (i) isolation and cultivation conditions can restrict the growth of strains; (ii) Lucas et al. (2019) analyzed the diversity of internal and external regions of *Cecropia-Azteca* whereas, in our work, we analyzed only samples of internal regions of *Cecropia-Azteca* such as ants, fungal spots, parenchyma, and eggs. Also, external areas, such as nest entrances and the branches, hosted microbiomes distinct from the workers’ chambers and the brood. In comparison, the carton colonized by ants, composed of macerated plant tissue and Chaetothyrialean fungi, also showed a smaller diversity than the external environment (Lucas et al., 2019). Interestingly, most of the genera we obtained were mainly from worker ants and fungal patches. These data obtained by culture-dependent methods are also in line with data obtained by culture-independent methods that showed a similarity in the structure of the *Azteca* ant community and the carton (fungal patches) (Lucas et al., 2019).

*Pseudomonas*, *Rhizobium*, *Methylobacterium, Pantoea* and *Streptomyces* were found in different parts of the sampled *Cecropia* and *Azteca* ants. The presence of these genera in ant-plant systems has been reported and could contribute to preparing vegetal substrate, acting in reinforcing the active transfer of nitrogen and playing a role in the defense of the domatia against diseases by limiting pathogen proliferation (Ruiz-González et al., 2019).

Potential diazotrophic microorganisms have already been recovered from different insect orders, such as Coleoptera, Blattodea, Diptera, Hemiptera and Hymenoptera (Behar et al., 2005; Morales-Jiménez et al., 2009; Pinto-Tomás et al., 2009; Bar-Shmuel et al., 2020). Bacteria belonging to the genus *Pantoea*, for example, have repeatedly been isolated from the leaf-cutting ants symbiosis sampled in different countries, such as Brazil, Argentina and Panama, and have been able to fix nitrogen in the fungus garden. The ants may have established symbiotic relationships with nitrogen-fixing microorganisms in order to provide the necessary demand for this element in the fungus garden (Pinto-Tomás et al., 2009).

Here, we found *Pseudomonas* isolates from the *Cecropia-Azteca* symbiosis carry out BNF *in vitro* and, thus, may be potentially involved in nitrogen fixation in the ant-plant interaction. This genus was suggested to be specifically associated with *Azteca* ants in recent work investigating microorganisms from *Cecropia-Azteca* in Panama (Lucas et al., 2019; Ruiz-González et al., 2019).

In addition to the possible nitrogen fixation function, we observed that *Pseudomonas* spp. strains may also play a role in protecting the colony, since they inhibited the growth of fungal entomopathogens and phytopathogens, including *P. clavispora* FB1 isolated from *Cecropia* sp. tree. Analysis of the microbial composition in different nests samples of *Azteca alfari* in *Cecropia* trees revealed that the absence of ants in the abandoned chambers showed significant growth of fungal pathogens. The presence of these fungi in the chambers and their absence in occupied ones, suggests that ants are important in regulating the development of such pathogens in their nests. Yet, this change is also related to the decrease of bacteria from phylum Proteobacteria, indicating that the microbiome can also play an important role in the control of pathogens (Lucas et al., 2019). This result supports our findings since proteobacteria, such as *Pseudomonas*, inhibited different pathogens.

Bioinformatic analysis revealed that *Pseudomonas* sp. ICBG1301 showed a BGC with 43% similarity to viscosin, thus this BGC could potentially be a member of the viscosin group of CLPs. Analysis on the adenylation and condensation domains showed that *Pseudomonas* sp. ICBG1301 is capable of producing viscosinamide. In addition, the second-module C_dual_-domain showed a mutation that potentially inactivated its epimerizing function, and a similar mutation might have occurred in the sixth module. Furthermore, the viscosinamide biosynthetic gene cluster is split into two *loci*, whereas the pseudodesmin BGC, its isomer, shows a contiguous gene cluster.

In order to confirm the structure of this product, we isolated and determined the structure of the CLP produced by *Cecropia-Azteca*-associated *Pseudomonas*. The CLP was unequivocally identified as viscosinamide, which is in accordance with our bioinformatic analyses. Furthermore, this result is also in agreement with the biological activity of its producer. All *Pseudomonas* strains showed antifungal activity in our binary screening and viscosinamide is a well-known antifungal agent. Its isomer, pseudodesmin A, does not show the same inhibitory properties, being selectively active against Gram-positive bacteria (Nielsen et al., 1999).

Our results have demonstrated that *Pseudomonas* spp. strains isolated from *Cecropia-Azteca* samples are active against ecological pathogens and show diazotroph activity. In addition, it was possible to gain insights on the biosynthesis of CLPs of the viscosin group. The cyclic lipodepsipeptide associated with the *Cecropia-Azteca* symbiosis was isolated and identified as viscosinamide. This compound has antifungal activity and can play an important ecological role in this symbiosis, since it was isolated from more than one strain and bacteria of the genus *Pseudomonas* are recurrently isolated from these samples. The fact that these strains seem to be relevant in different levels, providing nitrogen for the system and producing secondary metabolites that can inhibit the growth of potential pathogens, reinforces the importance of these bacteria for the *Cecropia-Azteca* symbiosis.

## Data Availability Statement

The datasets presented in this study can be found in online repositories. The names of the repository/repositories and accession number can be found below: https://www.ncbi.nlm.nih.gov/bioproject/ (Accession ID: PRJNA684609).

## Author Contributions

TF, CP, CC, and MP performed conceptualization. TF, CP, WM, and MP performed data curation and wrote the original draft. TF, CP, WM, CM, PA, PL, and MP performed formal analysis. TF, CP, WM, PA, MG, PL, CC, and MP performed investigation. TF, CP, WM, CM, PA, PL, and MP performed methodology. MP performed supervision. TF, CP, WM, CM, PA, MG, PL, CC, and MP performed visualization and wrote, review and editing the manuscript. All authors contributed to the article and approved the submitted version.

## Conflict of Interest

The authors declare that the research was conducted in the absence of any commercial or financial relationships that could be construed as a potential conflict of interest.
