# Supplementary material for: Insights Into the Ecological Role of Pseudomonas spp. in an Ant-plant Symbiosis
Source: Front Microbiol. 2021 Feb 1;12:621274. doi: 10.3389/fmicb.2021.621274 (PMC7882492; doi:10.3389/fmicb.2021.621274)
Supplement: Supplementary file 2 [file Presentation_1.pdf]

SUPPLEMENTARY INFORMATION

Insights into the ecological role of *Pseudomonas* spp. in an ant-plant symbiosis

Taise T. H. Fukuda[1†]; Camila F. Pereira[1†]; Weilan G. P. Melo[1]; Carla Menegatti[1]; Paulo H. M. Andrade[2]; Milton Groppo[3]; Paulo T. Lacava[2]; Cameron R. Currie[4]; Mônica T. Pupo[1*]

[†]These authors contributed equally to this work.

[1]School of Pharmaceutical Sciences of Ribeirão Preto, University of São Paulo, Ribeirão Preto, SP, Brazil.

[2]Laboratory of Microbiology and Biomolecules, Department of Morphology and Pathology, Center of Biological Sciences and Health, Federal University of São Carlos, São Carlos, SP, Brazil

[3]Laboratory of Plant Systematics, Department of Biology, Faculty of Philosophy, Sciences and Letters at Ribeirão Preto, University of São Paulo, Ribeirão Preto, SP, Brazil

[4]Department of Bacteriology, University of Wisconsin-Madison, Madison, WI, USA.

Table of contents

Figure S1. Isolation of the phytopathogen *Pestalotiopsis clavisora* from *Cecropia* leaves

Figure S2 - Heatmap of the biological activity of *Cecropia-Azteca* microbial strains against human pathogens, phytopathogens, and entomopathogens.

Figure S3 - Antifungal activity of *Pseudomonas* sp. ICBG1870 against *Pestalotiopsis clavispora* FB1.

Figure S4 - Phylogeny of 16S rRNA gene

Figure S5 - Phylogeny of condensation domains.

Figure S6 - Phylogeny of condensation domains.

Figure S7 - Phylogeny of condensation domains.

Figure S8 - Phylogeny of adenylation domains.

Figure S9 - HRESI-MS spectrum of viscosinamide.

Figure S10 - MALDI-MS/MS spectrum of viscosinamide.

Figure S11 - ^{1}H NMR spectrum of viscosinamide (CD_3COCD_3, 500 MHz).

Figure S12 - ^{1}H,^{1}H-COSY spectrum of viscosinamide (CD_3COCD_3, 500 MHz).

Figure S13 - *g*HSQC spectrum of viscosinamide (CD_3COCD_3, 500 MHz).

Figure S14 - *g*HMBC spectrum of viscosinamide (CD_3COCD_3, 500 MHz).

Table S1 - NMR spectroscopic data of viscosinamide (CD_3COCD_3).

Figure S15 - Chromatograms of viscosinamide products after hydrolysis and advanced Marfey derivatization.

Figure S16 - Chemical structures of viscosin, WLIP, viscosinamide A and massetolide A.

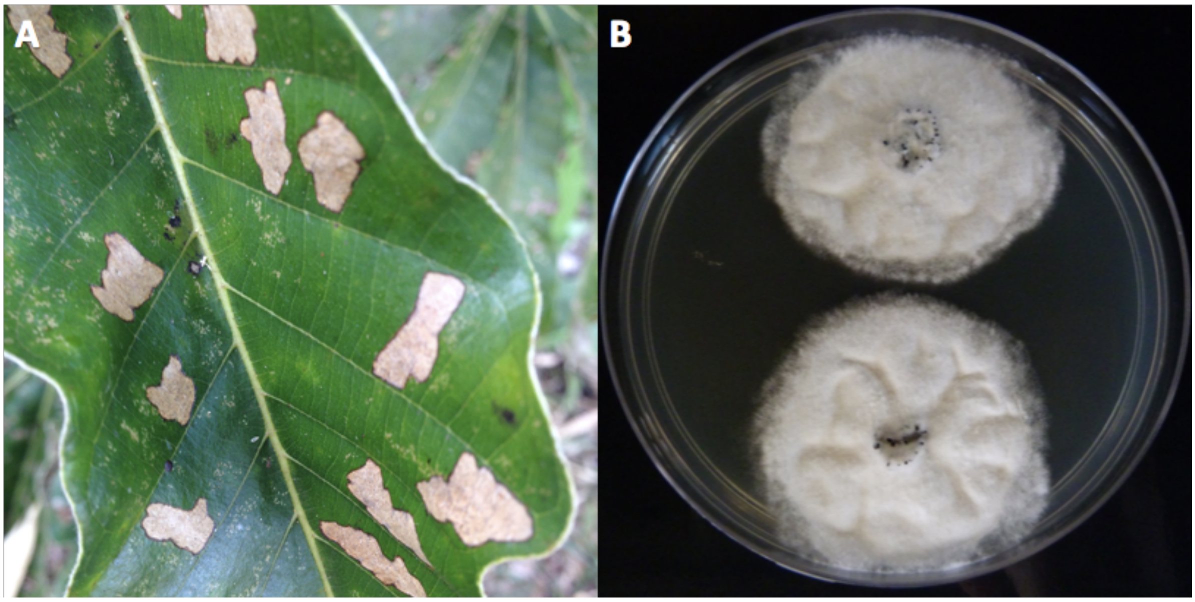

Figure S1. Isolation of the phytopathogen *Pestalotiopsis clavispora* from *Cecropia* leaves. **(A)** *Cecropia* sp. leaf contaminated with a fungal pathogen. **(B)** *P. clavispora* FB1 isolated from *Cecropia* sp.

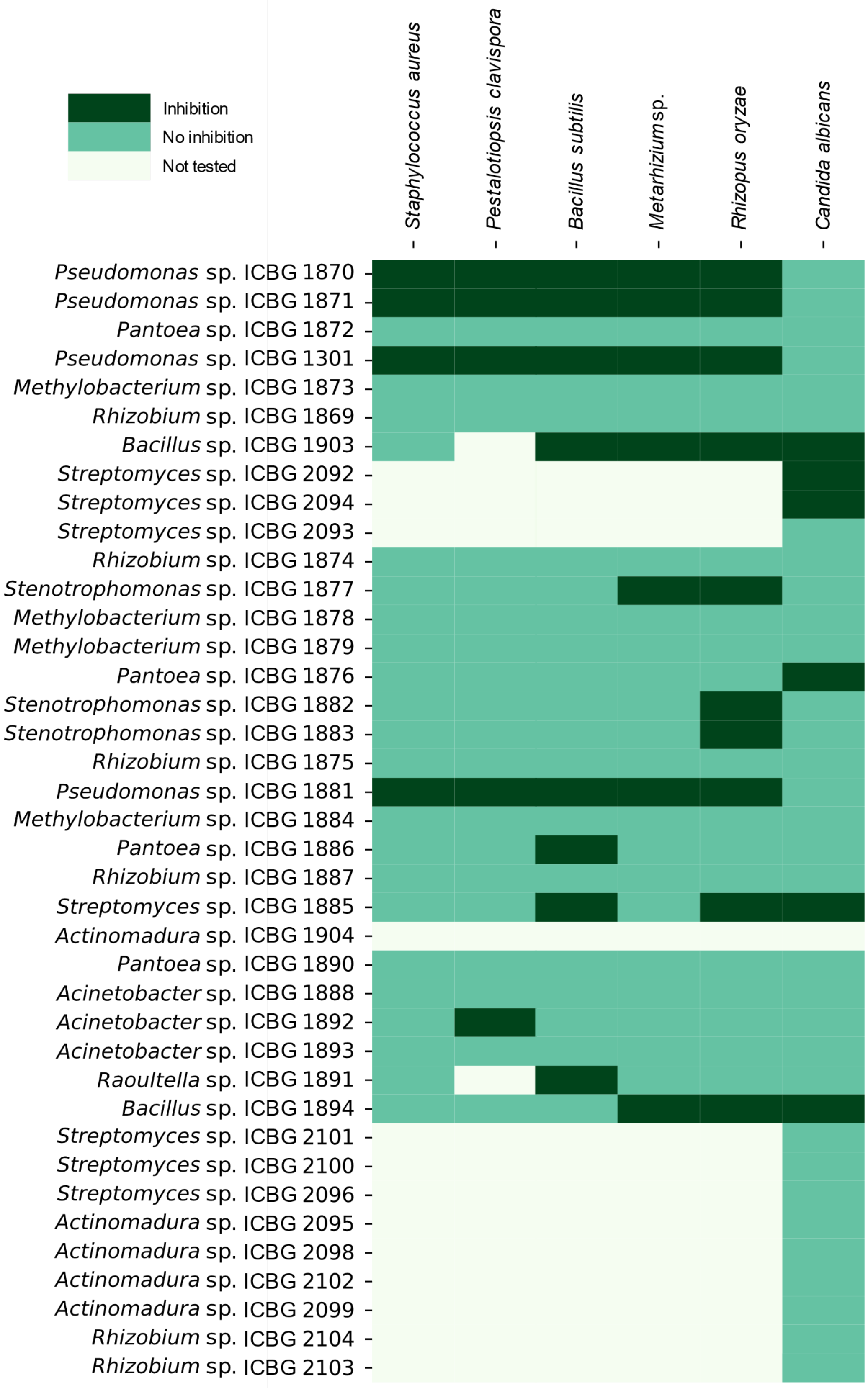

Figure S2. Heatmap of the biological activity of *Cecropia-Azteca* microbial strains against human pathogens, phytopathogens and an entomopathogen.

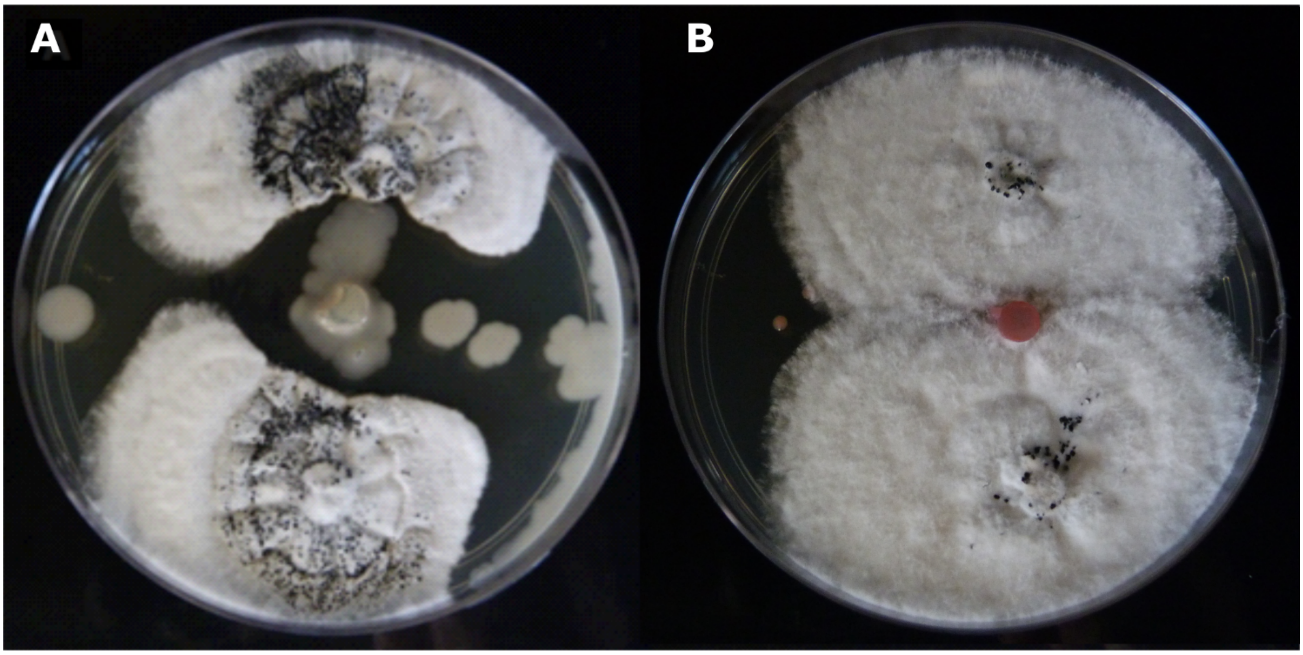

Figure S3. Antifungal activity of *Pseudomonas* sp. ICBG1870 against *Pestalotiopsis clavispora* FB1. **(A)** Inhibition zone of *Pseudomonas* sp. ICBG1870 against *P. clavispora*. **(B)** *Methylobacterium* sp. ICBG1884 against *P. clavispora,* negative control.

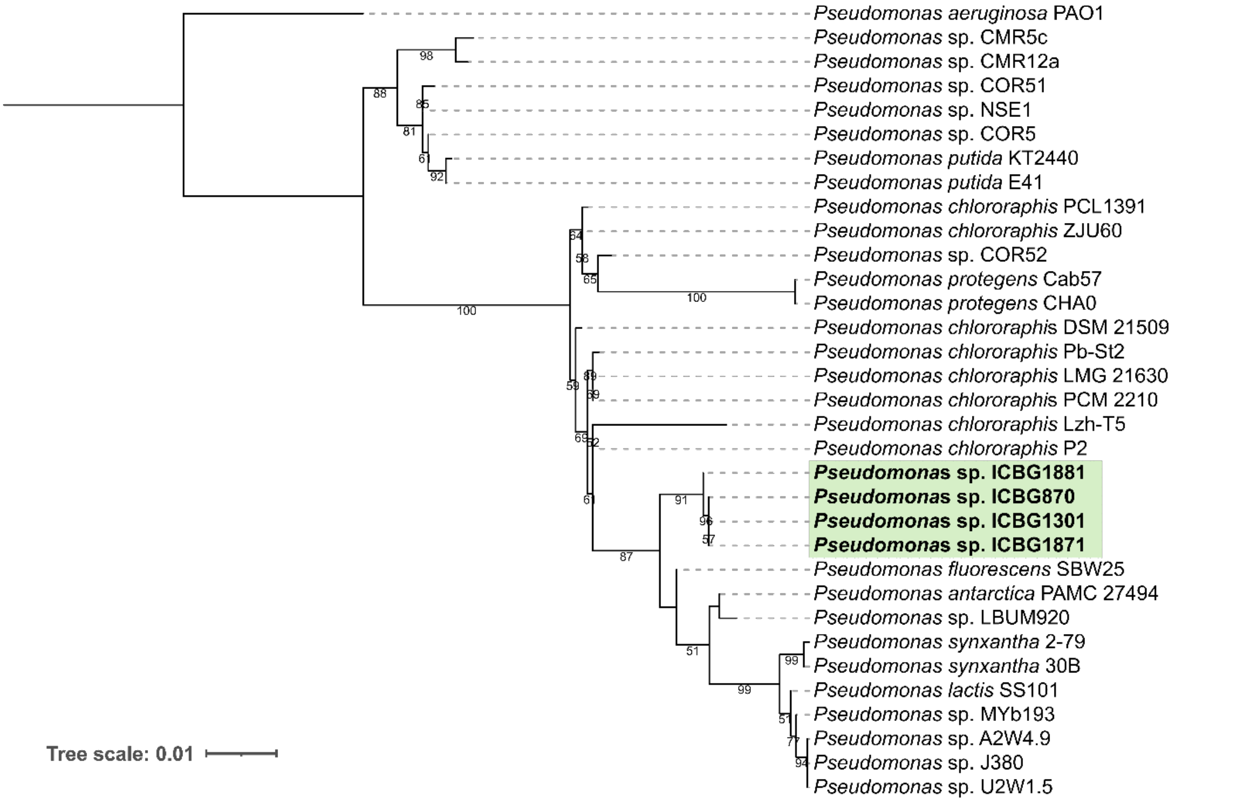

Figure S4. Phylogenetic analysis based on the 16S rRNA gene of viscosin-producers. The bootstrap support values are based on 1000 bootstrap replicates. The phylogeny was reconstructed using IQ-TREE, using the TPM3+F+R2 model of nucleotide substitution and including *P. aeruginosa* PAO1 as outgroup.

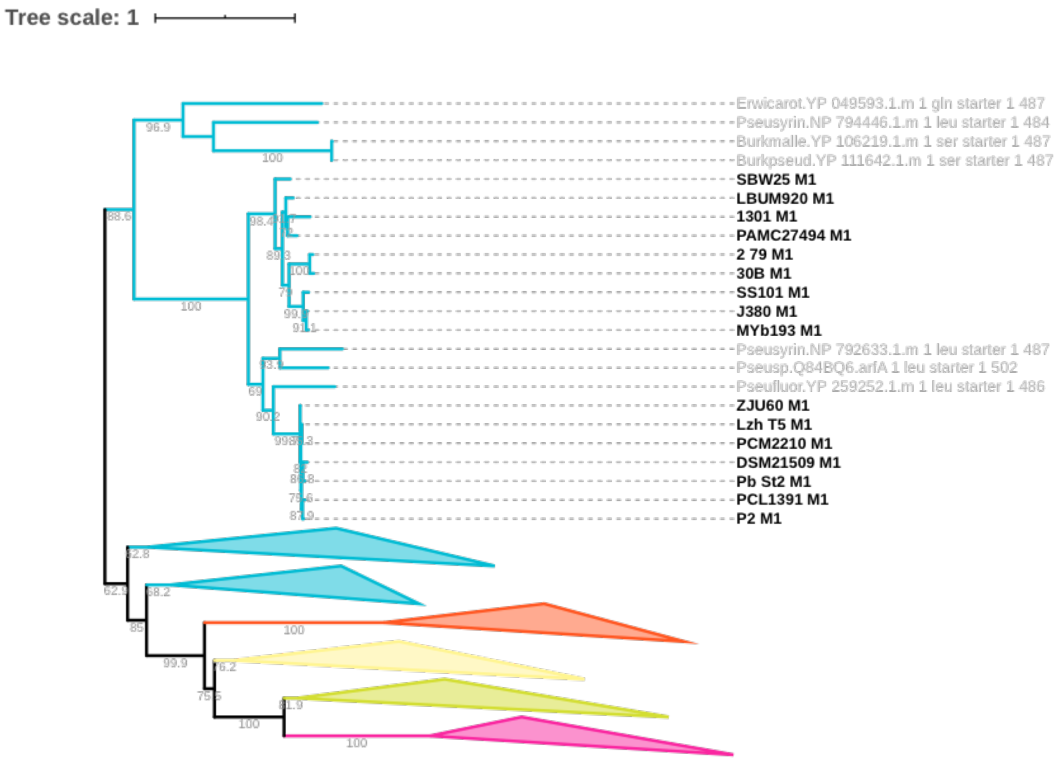

Figure S5. Phylogeny of condensation domains. The phylogeny was reconstructed using IQ-TREE, using the LG+F+R10 amino acid substitution model. Support values are based on 1000 bootstrap replicas. Blue: clade that comprises $C_{starter}$ modules; orange: Cyc; yellow: LCL; green: DCL; pink: C_{dual}. The clade that comprises the condensation domains of module 1 viscosins-producers is highlighted in bold. Other clades were collapsed.

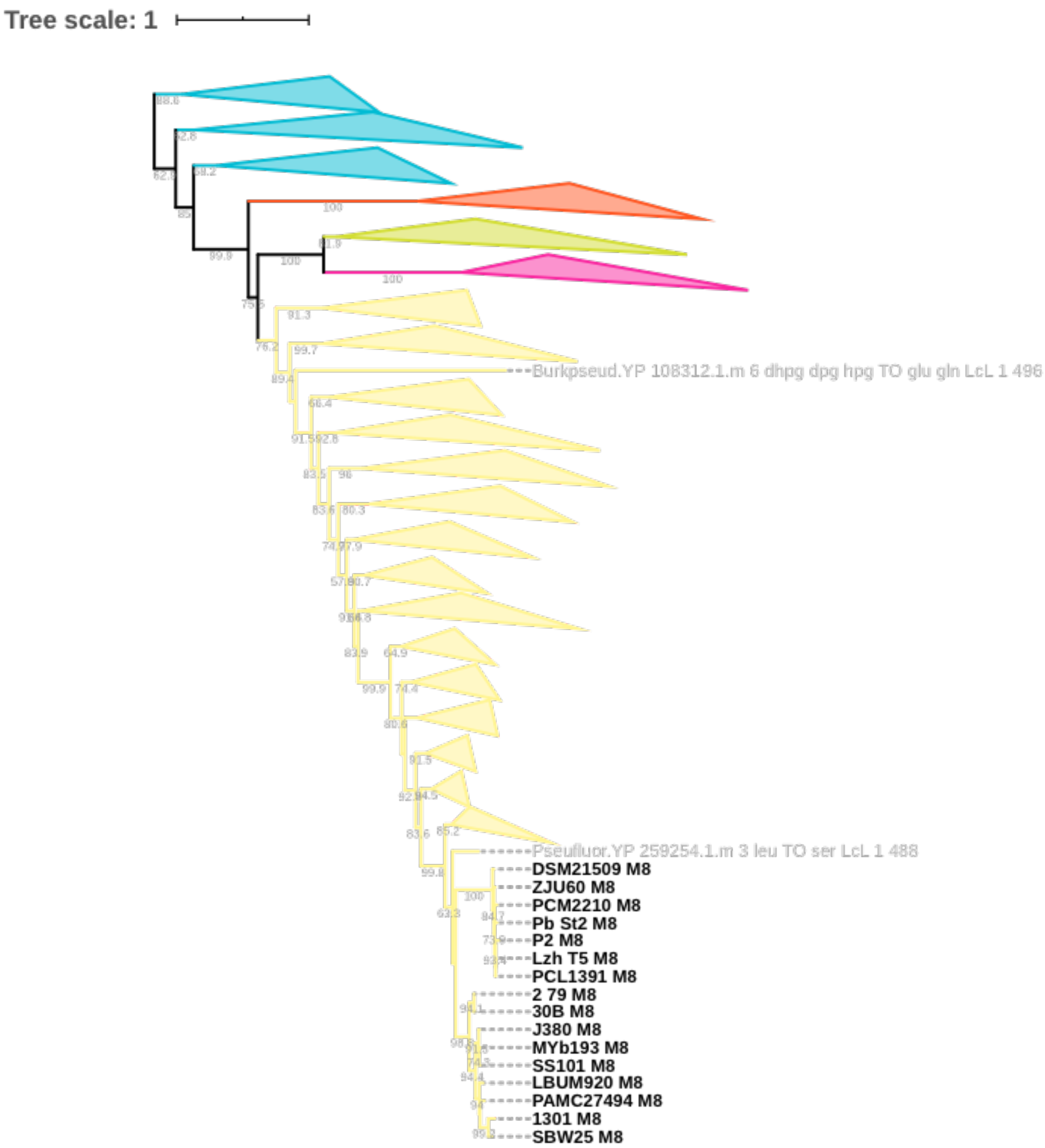

Figure S6. Phylogeny of condensation domains. The phylogeny was reconstructed using IQ-TREE, using the LG+F+R10 amino acid substitution model. Support values are based on 1000 bootstrap replicas. Blue: clade that comprises $C_{starter}$ modules; orange: Cyc; yellow: LCL; green: DCL; pink: C_{dual}. The clade that comprises the condensation domains of module 8 viscosins-producers is highlighted in bold. Other clades were collapsed.

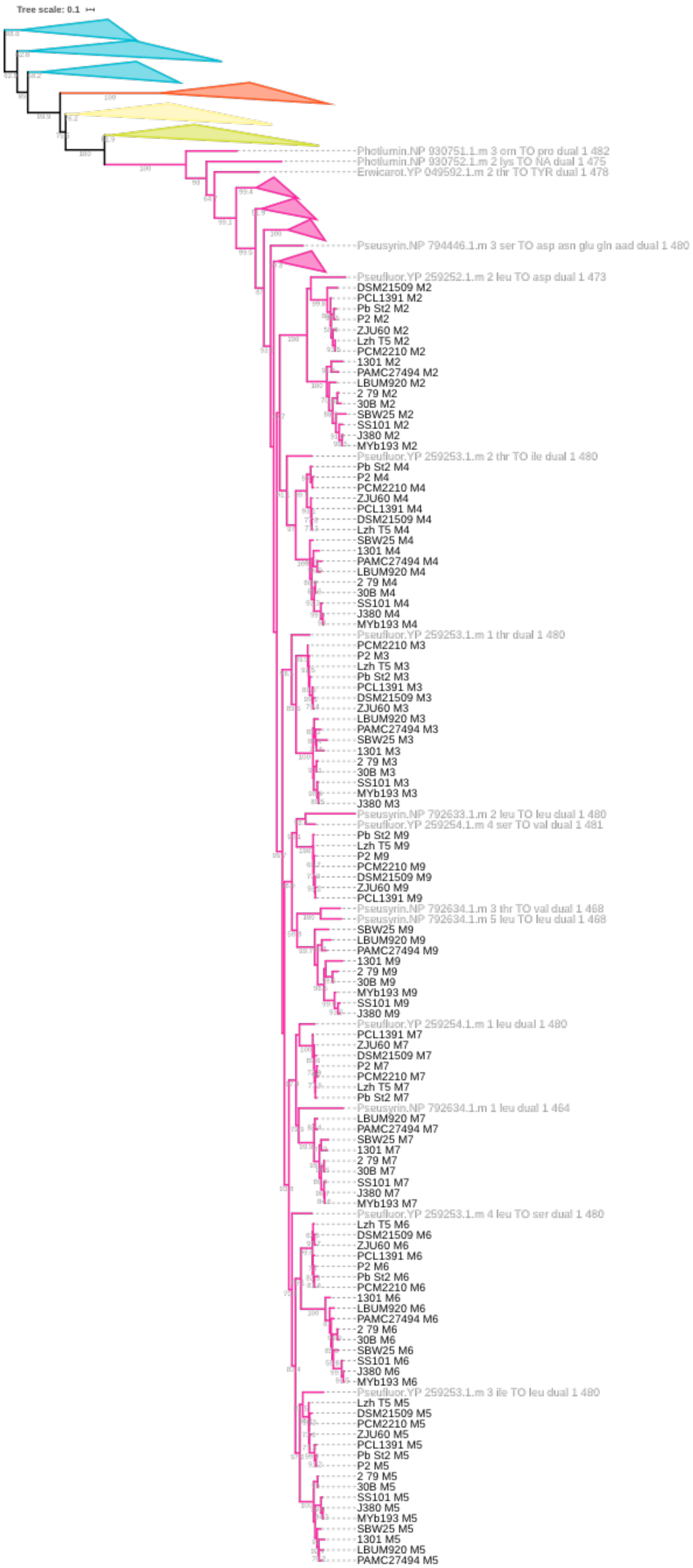
Tree scale: 0.1
Photlumin.NP 930751.1.m 3 orn TO pro dual 1 482
Photlumin.NP 930752.1.m 2 lys TO NA dual 1 475
Erwicarot.YP 049592.1.m 2 thr TO TYR dual 1 478
Pseusyrin.NP 794446.1.m 3 ser TO asp asn glu gln aad dual 1 480
Pseufluor.YP 259252.1.m 2 leu TO asp dual 1 473
DSM21509 M2
PCL1391 M2
Pb St2 M2
P2 M2
ZJU60 M2
Lzh T5 M2
PCM2210 M2
1301 M2
PAMC27494 M2
LBUM920 M2
2 79 M2
30B M2
SBW25 M2
SS101 M2
J380 M2
MYb193 M2
Pseufluor.YP 259253.1.m 2 thr TO ile dual 1 480
Pb St2 M4
P2 M4
PCM2210 M4
ZJU60 M4
PCL1391 M4
DSM21509 M4
Lzh T5 M4
SBW25 M4
1301 M4
PAMC27494 M4
LBUM920 M4
2 79 M4
30B M4
SS101 M4
J380 M4
MYb193 M4
Pseufluor.YP 259253.1.m 1 thr dual 1 480
PCM2210 M3
P2 M3
Lzh T5 M3
Pb St2 M3
PCL1391 M3
DSM21509 M3
ZJU60 M3
LBUM920 M3
PAMC27494 M3
SBW25 M3
1301 M3
2 79 M3
30B M3
SS101 M3
MYb193 M3
J380 M3
Pseusyrin.NP 792633.1.m 2 leu TO leu dual 1 480
Pseufluor.YP 259254.1.m 4 ser TO val dual 1 481
Pb St2 M9
Lzh T5 M9
P2 M9
PCM2210 M9
DSM21509 M9
ZJU60 M9
PCL1391 M9
Pseusyrin.NP 792634.1.m 3 thr TO val dual 1 468
Pseusyrin.NP 792634.1.m 5 leu TO leu dual 1 468
SBW25 M9
LBUM920 M9
PAMC27494 M9
1301 M9
2 79 M9
30B M9
MYb193 M9
SS101 M9
J380 M9
Pseufluor.YP 259254.1.m 1 leu dual 1 480
PCL1391 M7
ZJU60 M7
DSM21509 M7
P2 M7
PCM2210 M7
Lzh T5 M7
Pb St2 M7
Pseusyrin.NP 792634.1.m 1 leu dual 1 464
LBUM920 M7
PAMC27494 M7
SBW25 M7
1301 M7
2 79 M7
30B M7
SS101 M7
J380 M7
MYb193 M7
Pseufluor.YP 259253.1.m 4 leu TO ser dual 1 480
Lzh T5 M6
DSM21509 M6
ZJU60 M6
PCL1391 M6
P2 M6
Pb St2 M6
PCM2210 M6
1301 M6
LBUM920 M6
PAMC27494 M6
2 79 M6
30B M6
SBW25 M6
SS101 M6
J380 M6
MYb193 M6
Pseufluor.YP 259253.1.m 3 ile TO leu dual 1 480
Lzh T5 M5
DSM21509 M5
PCM2210 M5
ZJU60 M5
PCL1391 M5
Pb St2 M5
P2 M5
2 79 M5
30B M5
SS101 M5
J380 M5
MYb193 M5
SBW25 M5
1301 M5
LBUM920 M5
PAMC27494 M5

Figure S7. Phylogeny of condensation domains. The phylogeny was reconstructed using IQ-TREE, using the LG+F+R10 amino acid substitution model. Support values are based on 1000 bootstrap replicas. Blue: clade that comprises $C_{starter}$ modules; orange: Cyc; yellow: LCL; green: DCL; pink: C_{dual}. The clade that comprises the C_{dual} domains of viscosins-producers is highlighted in bold. Other clades were collapsed.

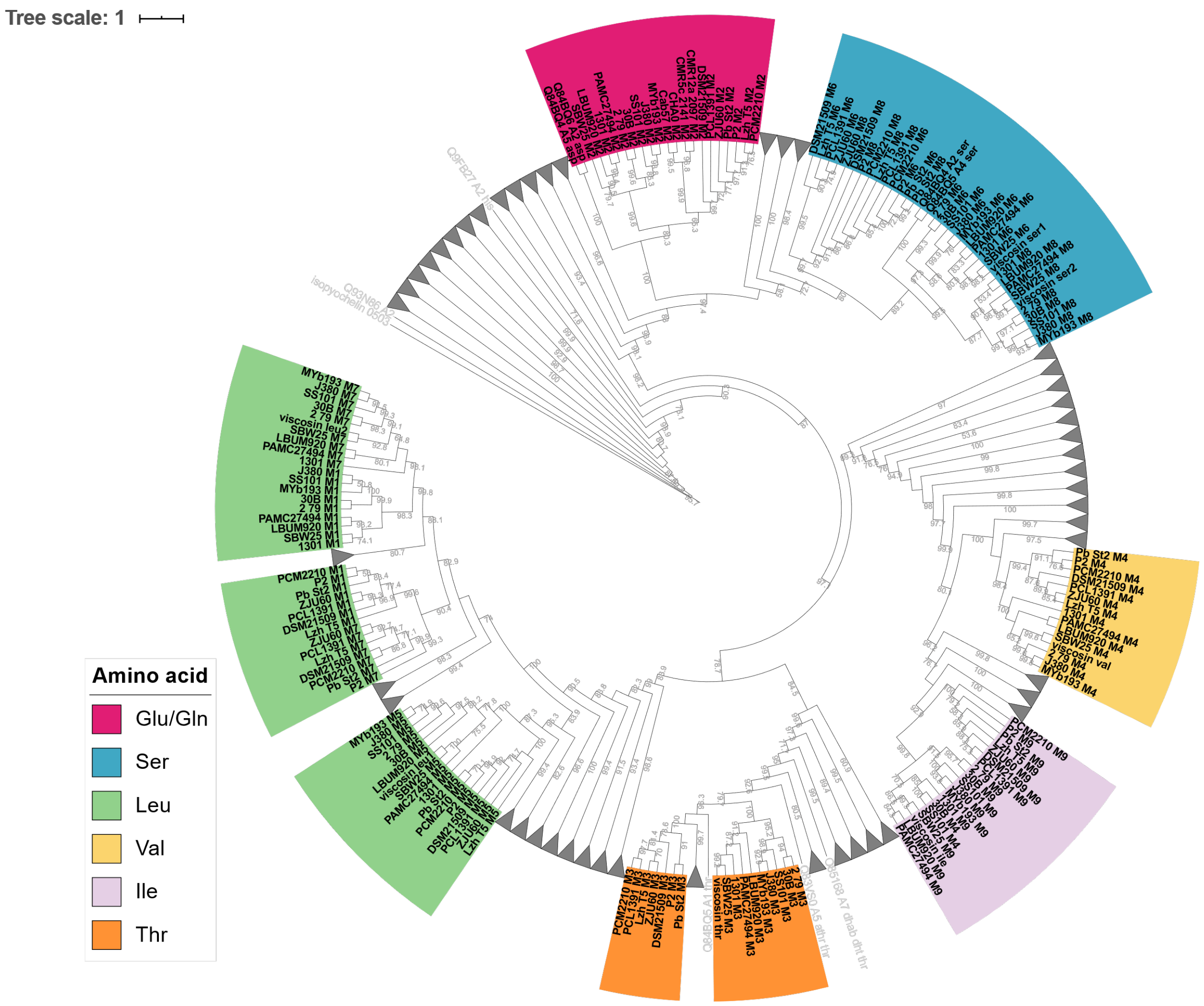

Figure S8. Phylogeny of adenylation domains. The phylogeny was reconstructed using IQ-TREE, using the LG+F+R10 amino acid substitution model. Support values are based on 1000 bootstrap replicas. In pink are domains responsible for recruiting Glu/Gln; in blue, Ser; in green, Leu; in yellow, Val; in lilac, Ile; in orange, Thr. Other clades were collapsed.

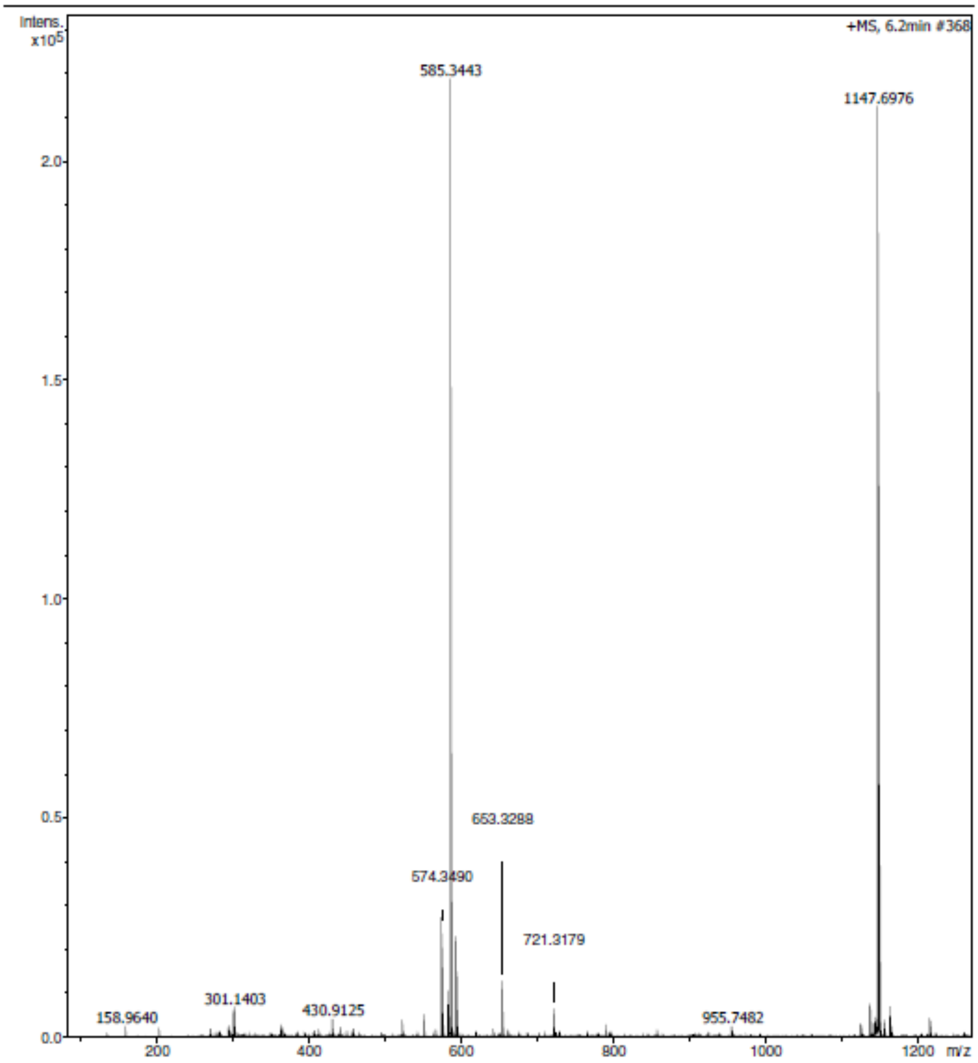

Figure S9. HRESI-MS spectrum of viscosinamide.

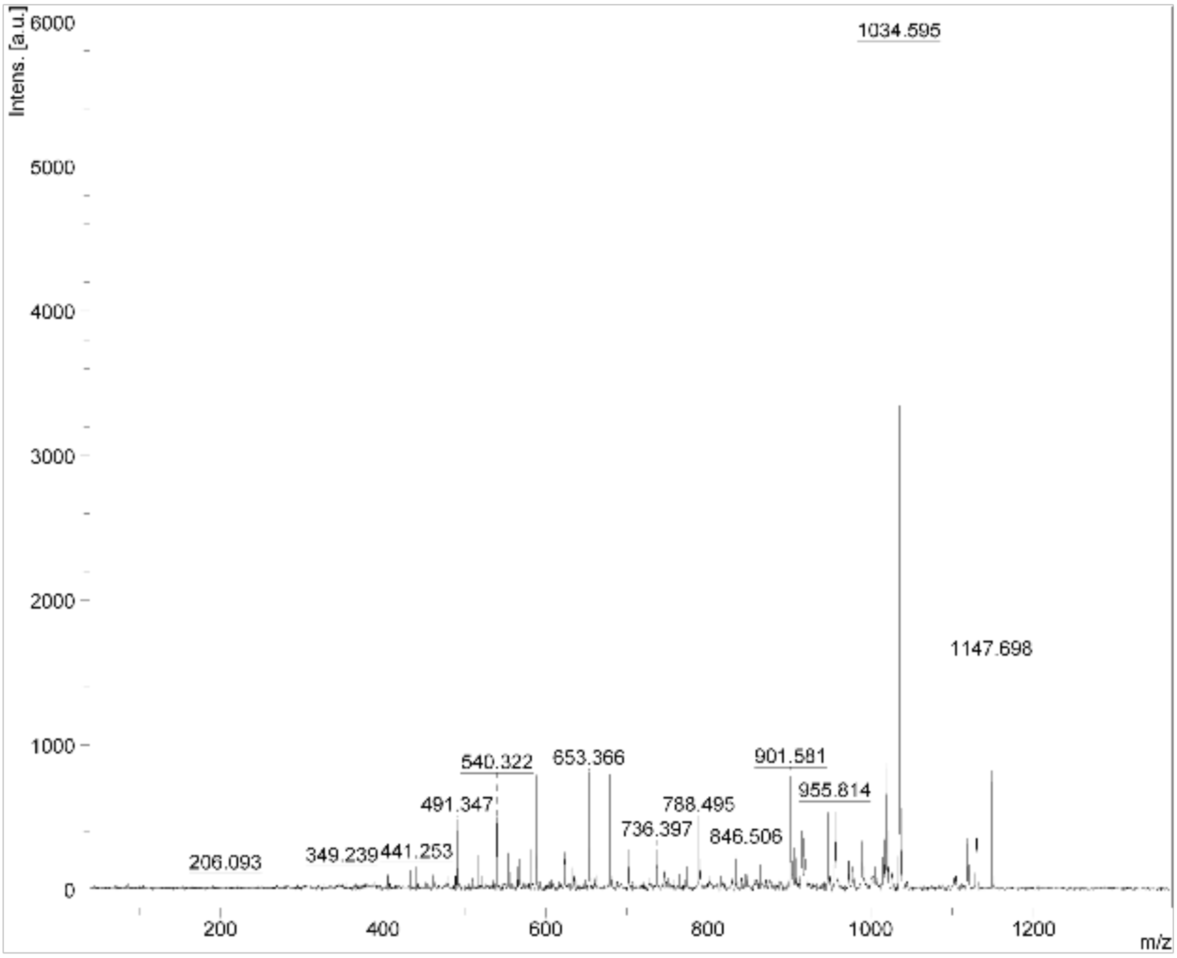

Figure S10. MALDI-MS/MS spectrum of viscosinamide.

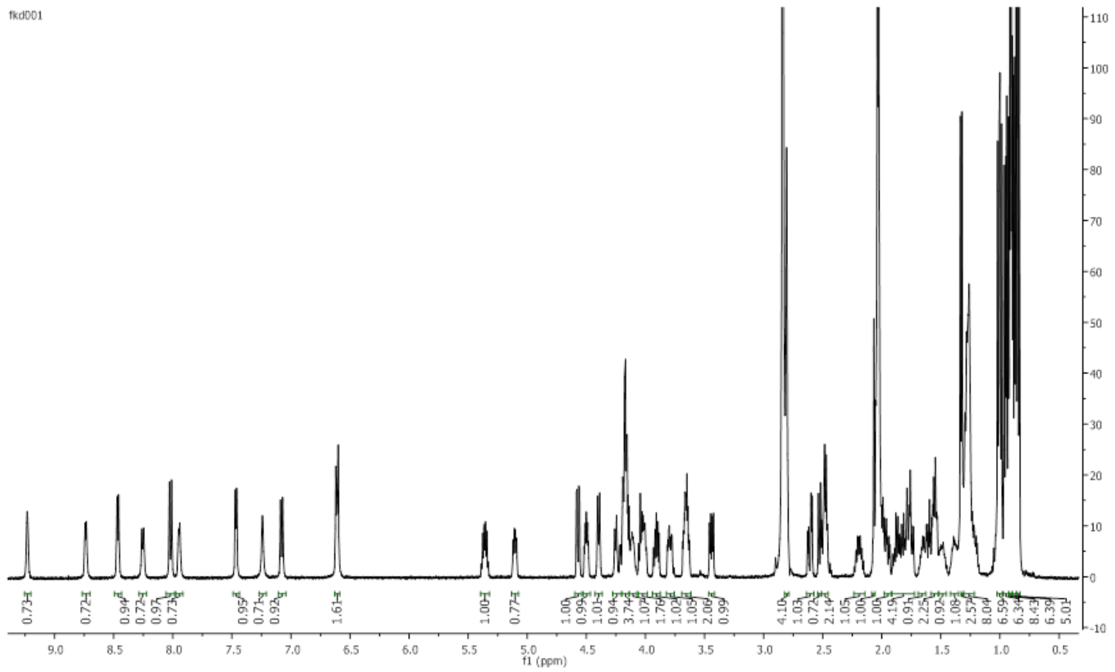

Figure S11. ^{1}H NMR spectrum of viscosinamide (CD_3COCD_3, 500 MHz).

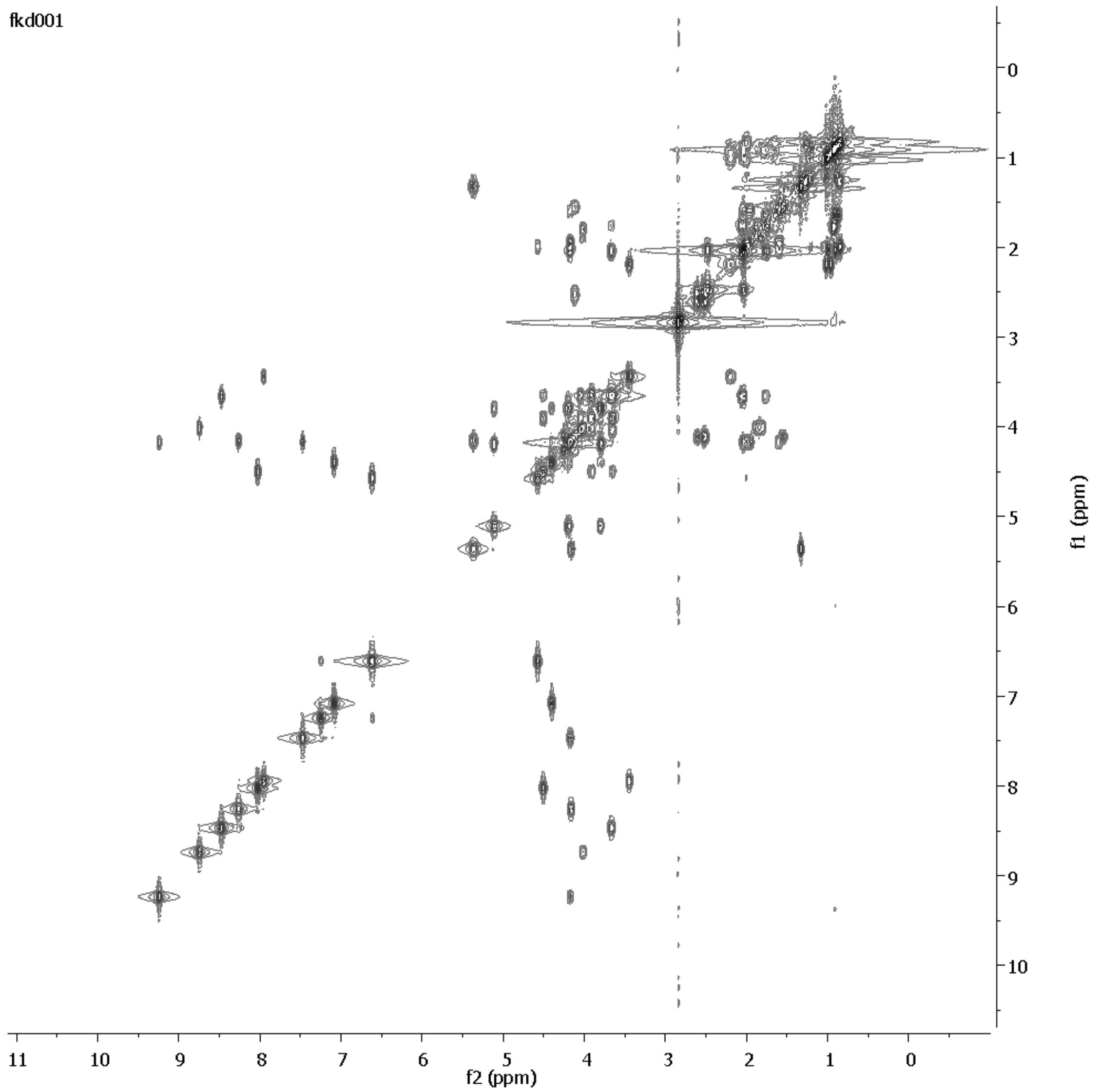

Figure S12. $^{1}H,^{1}H$-COSY spectrum of viscosinamide (CD_3COCD_3, 500 MHz).

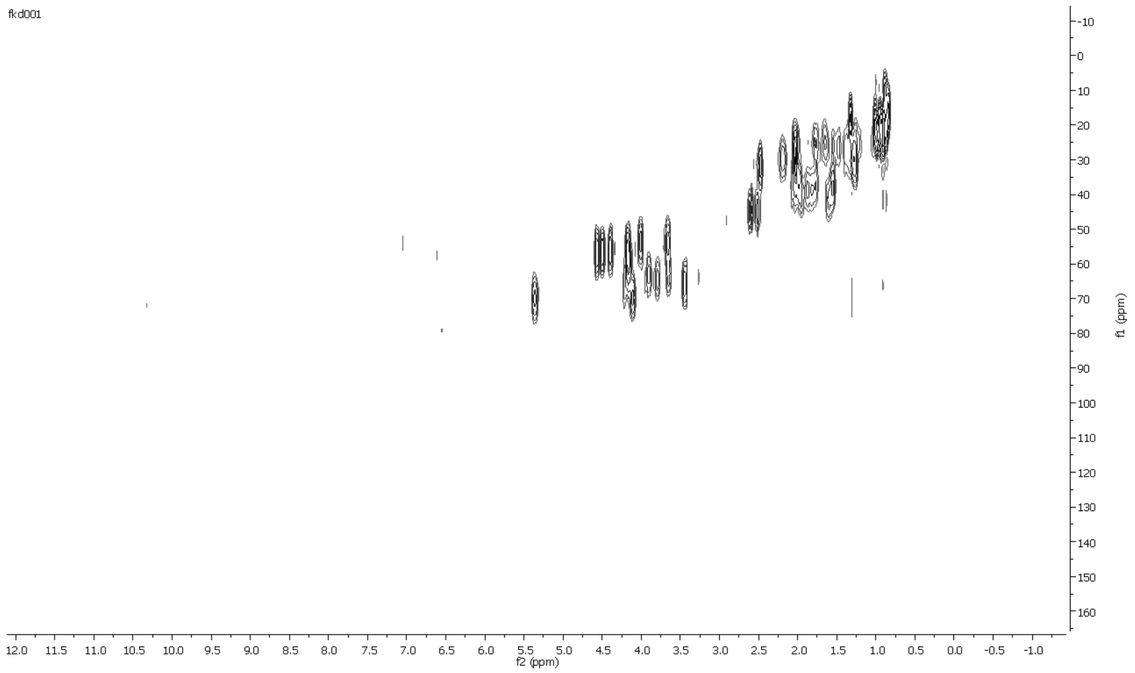

Figure S13. *g*HSQC spectrum of viscosinamide (CD_3COCD_3, 500 MHz).

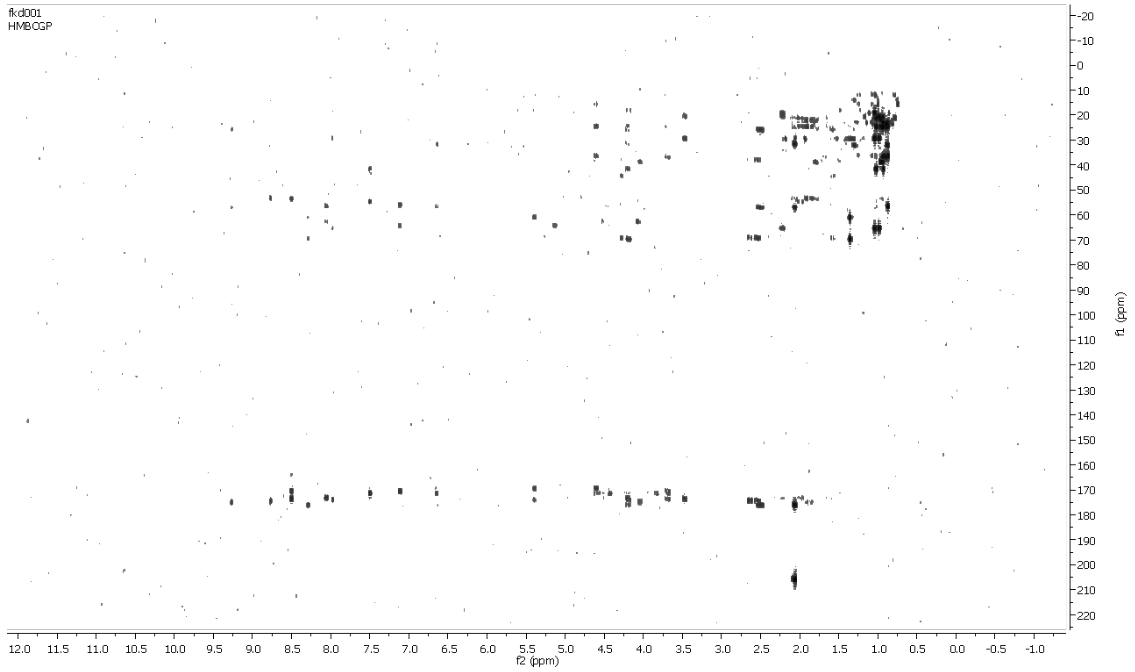

Figure S14. *g*HMBC spectrum of viscosinamide (CD_3COCD_3, 500 MHz).

Table S1. NMR spectroscopic data of viscosinamide (CD_3COCD_3).

Amino acid	Position	δC	δH	Multiplicity (Hz)	COSY	HMBC
Fatty acid	1	174.7	--			
	2	44.7	2.61<br>2.53	dd (14.23; 4.04)<br>d (9.47)	3<br>3	1, 3<br>1, 3, 4, 5
	3	69.7	4.11	m	4	
	4	38.3	1.55	m	5	
	5	26.3	1.48	m		
	6	n.a.	n.a.			
	7	n.a.	n.a.			
	8	n.a.	n.a.			
	9	n.a.	n.a.			
	10	n.a.	n.a.			
	11	n.a.	n.a.			
Leu1*	12		8.74	d (5.88)	13	13, 14
	13	53.4	3.99	m	12, 15	15,14
	14	174.9	--			
	15	38.9	1.87	m	13, 16	16, 17, 13,
	16	25.3	1.77	m	15, 17	13, 17
	17	22.3	0.91	m	16	16, 13
	18	21.3	0.91	m	16	16, 13
Gln2	19	--	8.25	d (7.10)	20	20
	20	58.3	4.15	m	22	23, 21,
	21	176.6	--			
	22	28.7	2.02	m	20, 23	23,20,24
	23	31.7	2.48	dt (11.10; 5.92)	22	24, 20,
	24	176.5	--			
	25	--	7.24/6.61	s		
Thr3	26		9.23	br s	27	27, 28
	27	57.0	4.17	m	29	28, 21,30, 29
	28	174.5	--			
	29	71	5.36	dq (12.00; 5.89)	27, 30	28, 63
	30	17.9	1.32	d (6.00)	29	
Val4	31	--	7.95	d (5.21)	32	32, 34, 33
	32	65.8	3.44	dd (11.08; 5.63)	34	35, 34,
	33	174.1	--			
	34	29.7	2.19	m	35,36	33,35, 32
	35	20.3	1.01	m	34	36
	36	19.5	0.96	m	34	35,34,32
Leu5*	37	--	8.47	d (6.75)	38	38, 33, 39,
	38	54	3.67	m	40	41, 39, 33
	39	171.1	--			
	40	38.8	1.77	m	38,41	38, 40, 43
	41	25.6	1.62	m	42,43	43
	42	21.3	0.92	m	41	43, 41
	43	21.8	0.94	m	41	41, 40
Ser6	44	--	07.08	d (5.69)	45	45, 47
	45	56.4	4.4	m	44,47	46,47
	46	171.9	--			
	47	64.5	3.79	m	45,48	46
	48	--	5.11	dd (8.98; 5.59)	47	47
Leu7*	49	--	7.47	d (8.45)	50	46, 52
	50	56.2	4.16	dt (8.42; 2.43)	49	52, 51, 46

	51	173.6	--			
	52	41.6	1.60	m	53	50
	53	36.1	1.98	m		50, 51
	54	16	0.84	m		50
	55	16	0.85	m		50
Ser8	56	--	8.02	d (9.14)	57	57, 51,59
	57	56.7	4.50	dt (8.88; 4.45)	56,59	59,58, 51
	58	171.7	--			
	59	62.4	3.65	m	60, 57	58
	60	--	4.04	m	59	59
Ile9	61	--	6.61	d (9.99)	62	62,58,63
	62	56.7	4.57	dd (10.15; 3.03)	61,64	64,65,66,63,58
	63	170.1	--			
	64	36.1	1.98	m	62, 65,66	66,67
	65	16	0.85	m	64	64, 66, 67, 62
	66	25.1	1.6	m	67,64	62
	67	21.3	0.94	m	66	66

* Interchangeable.

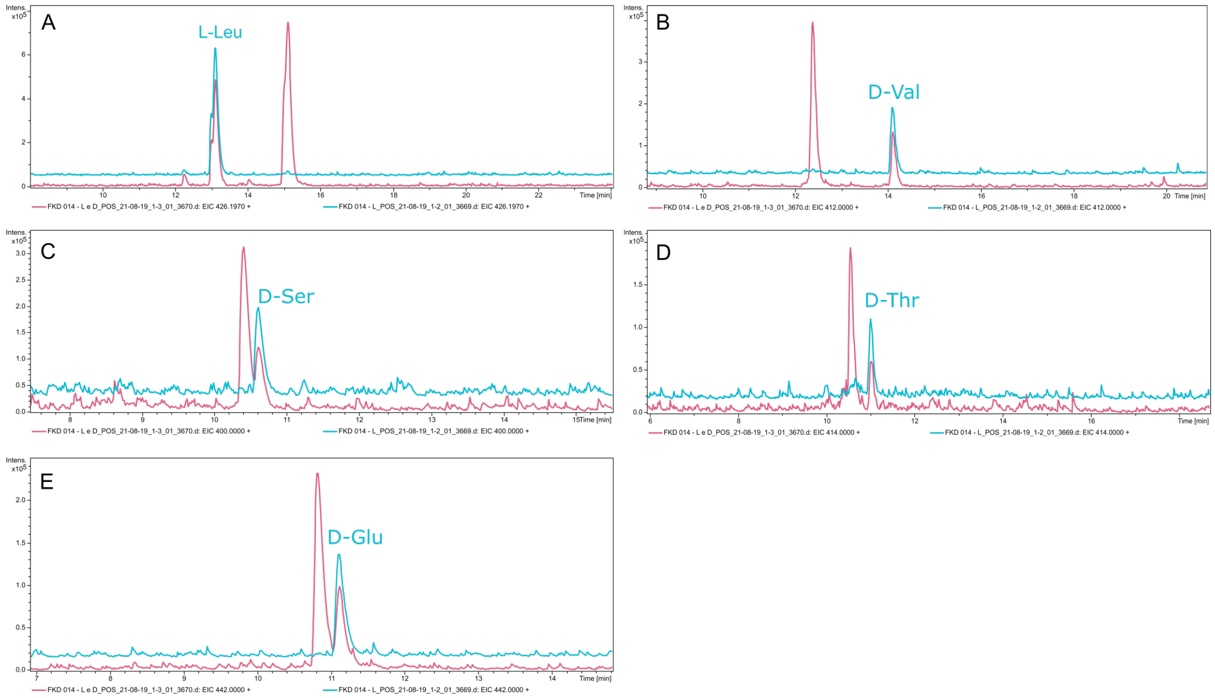

Figure S15. Chromatograms of viscosinamide products after hydrolysis and advanced Marfey derivatization. In blue, the chromatograms of the product of the reaction with L-FDLA are represented, and in pink, with L, D-FDLA. (A) EIC *m/z* 426.45, corresponding to L-Leu. (B) EIC *m/z* 412.43, corresponding to D-Val. (C) EIC *m/z* 400.37, corresponding to D-Ser. (D) EIC *m/z* 414.4, corresponding to D-Thr. (E) EIC *m/z* 442.41, corresponding to D-Glu.

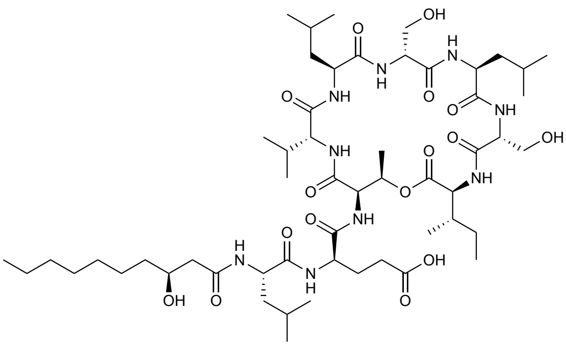

VISCOSIN

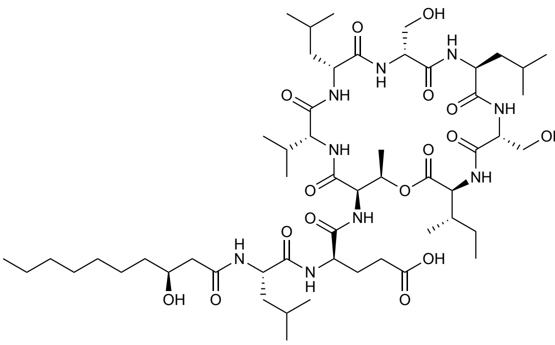

WLIP

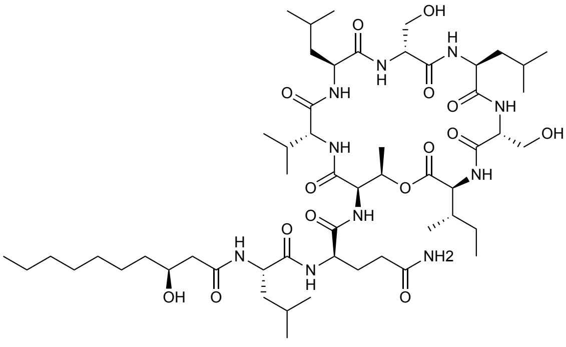

VISCOSINAMIDE A

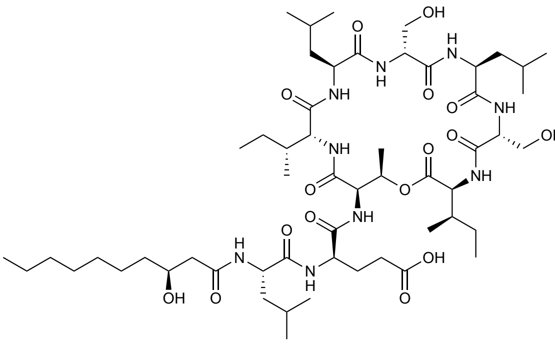

MASSETOLIDE A

Figure S16. Chemical structures of viscosin, WLIP, viscosinamide A and massetolide A.
